# Perceptions of coastal vegetated ecosystems: A systematic review across geographical and sectoral dimensions

**DOI:** 10.1007/s13280-025-02193-x

**Published:** 2025-05-29

**Authors:** Jay Mar D. Quevedo, Michelle Ann Miller, Dixon T. Gevaña, Danny Marks, Daniel A. Friess, Prayoto Tonoto, David Taylor

**Affiliations:** 1 Asia Research Institute, National University of Singapore, AS8, #07-45, 10 Kent Ridge Crescent, Singapore, 119260 Singapore; 2 College of Forestry and Natural Resources, University of the Philippines, Tamesis Bldg. Martin Reyes St., 4031 Los Baños, Philippines; 3 School of Law and Government, Dublin City University, DCU Glasnevin Campus, Dublin 9, Ireland; 4 Department of Earth and Environmental Sciences, Tulane University, New Orleans, LA 70118 USA; 5 Riau Provincial Environment and Forestry Office, Jl. Jenderal Sudirman No. 468, Pekanbaru City, Riau 28126 Indonesia; 6 Department of Geography, Faculty of Arts and Social Sciences, National University of Singapore, AS2-03-01, 1 Arts Link, Singapore, 117568 Singapore

**Keywords:** Blue carbon, Content analysis, Governance, Management, Sector-level perceptions

## Abstract

Coastal vegetated ecosystems such as mangrove forests, seagrass meadows, and tidal marshes provide a wide array of ecosystem services. They also play a vital role in climate change mitigation through carbon sequestration. However, they are among the most threatened ecosystems globally. This study addresses a key knowledge gap by conducting a systematic review of academic literature on sectoral perceptions of these ecosystems. Through content analysis, we identified common research themes across regions and examined how members of the public, private, and civil society sectors perceive coastal vegetated ecosystems. The results reveal regional differences: Asian countries tend to emphasize utilization and economic benefits, while North America and Europe focus more on conservation and management. Several studies explored perceptions of climate change mitigation. Our findings highlight gaps in perception between the private and government agencies. Understanding these diverse sectoral perspectives can inform policy interventions to enhance conservation efforts and strengthen governance strategies.

**Supplementary Information:**

The online version contains supplementary material available at 10.1007/s13280-025-02193-x.

## Introduction

Coastal vegetated ecosystems such as mangrove forests, seagrass meadows, and tidal marshes provide diverse ecosystem services, including food, protection against coastal hazards, and support for a variety of cultural benefits (Himes-Cornell et al. 2018; Quevedo and Kohsaka 2024). These habitats have gained scientific recognition for their significant role in mitigating climate change by sequestering carbon (Macreadie et al. 2021), also known as “blue carbon” (Nellemann et al. 2009; Hilmi et al. 2021). In this context, numerous studies have mapped the areal extent of coastal vegetated ecosystems, quantified carbon stocks and fluxes, and monetized their carbon value (e.g., Ruiz-Frau et al. 2017; Ho and Mukul 2021). Recent advancements have further refined these efforts through remote sensing for cost-effective blue carbon accounting (Malerba et al. 2023) and the expansion of blue carbon markets (Friess et al. 2022). At the same time, social and policy-driven studies continue to evolve, highlighting the role of various actors in coastal vegetated ecosystems research and conservation (Quevedo et al. 2023).

Evolving scientific research on coastal vegetated ecosystems offers insights into sustainable management and policy development. However, governance challenges persist, particularly at the local level, where stakeholders can be directly involved in the management and/ or exploitation on a day-to-day basis (Merk et al. 2022). At the national level, there are other challenges such as financial constraints, overlapping directives and policies, and competing priorities within and between government departments. These issues can lead to conflicting management plans and interventions due to different ecological knowledge bases and value systems. Another challenge lies in power asymmetries, where more influential actors and institutions exert greater control over coastal policies and activities (Thompson 2018; Ayostina et al. 2022; Quevedo et al. 2024). Addressing these challenges requires a deeper understanding of how various stakeholders interact with coastal vegetated ecosystems and each other, and how sector-level perceptions vary over time.

Perception can be defined as the way individuals observe, understand, interpret, and evaluate objects, actions, or policies (Bennett 2016). Research on stakeholders’ perceptions recognizes their variation according to the influence of multiple factors, including (but not limited to) socioeconomic conditions, resource access, and personal connections and experiences of the environment (Jefferson et al. 2015; Bennett 2016). Understanding how different stakeholders perceive the environment, particularly the ways in which people value and connect with an ecosystem in accordance with its capacity to provide services that meet their own needs (Jefferson et al. 2015; de Souza Queiroz et al. 2017), is a basis for inclusive decision-making processes linked to the formulation and implementation of new policies and programs (Bennett et al. 2021). In this regard, studies have been conducted to investigate people’s perceptions to determine the importance of various implementation and research issues. For example, Ayostina et al. (2022) utilized social network analysis to map actors, their perceptions, and the patterns of information exchanged within networks, identifying potential barriers in blue carbon governance and policy development. Similarly, the services provided by coastal vegetated ecosystems, including blue carbon, have been investigated across various target sites (Quevedo et al. 2020; Arumugan et al. 2021).

Our systematic review focuses on perceptions of selected coastal vegetated ecosystems globally to analyze: (i) recurring research themes across geographical regions and (ii) how sector-level stakeholders are understood to perceive these habitats. The first focus draws inspiration from similar works that illustrate a global view of the research landscape and identify trends in perceived themes within and between countries (e.g., Jefferson et al. 2021). At local, national, regional, and global scales, perceptions among stakeholders may vary within and between countries. This variation can be attributed, for instance, to national institutional capacities, research foci, availability of funds for research, and environmental and cultural factors (Bertram and Merk 2020; Jefferson et al. 2021; Low et al. 2024). Understanding the geographical distribution of studies provides insights into varying levels of awareness and research, in this case, on perceptions of coastal vegetated ecosystems. The second focus builds on the nascent literature into sectoral perceptions of mangrove forests, seagrass meadows, and tidal marshes, including recognition of various ecosystem services, socio-cultural and economic valuations, traditional and Indigenous viewpoints, and associated governance considerations.

In this study, we focus on three sectors that play an important role in the governance of coastal vegetated ecosystems, namely the public sector (e.g., government agencies, village leaders), private sector (e.g., business representatives, landowners), and civil society (e.g., community-based, non-government, and Indigenous people’s organizations) (Thompson 2018; Ayostina et al. 2022; Quevedo et al. 2024). Understanding different sector-level perceptions of coastal vegetated ecosystems is important for informing their integrated governance (Dahouh-Guebas et al. 2020; Lukman et al. 2021). For example, research on perceptions could capture the less visible dimensions of complementary and/or competing values among relevant stakeholders that in turn inform sector-level policy choices and interventions into mangroves (Thompson 2018; Miller and Tonoto 2023).

By synthesizing these two foci, our review of sector-level perceptions of mangrove forests, seagrass meadows, and tidal marshes aims to provide a foundation for informing future research and policy developments and interventions, which are currently understudied (Pang et al. 2024; Sun et al. 2024). This review highlights research opportunities to contribute to recognition of sectoral perceptions of coastal vegetated ecosystems. We argue that building understanding of sector-level perceptions is important for capturing the diversity of interests in these habitats and can be a precursor for developing more integrated and socially inclusive forms of governance.

## Materials and methods

### Data collection and screening process

**Fig. 1:**
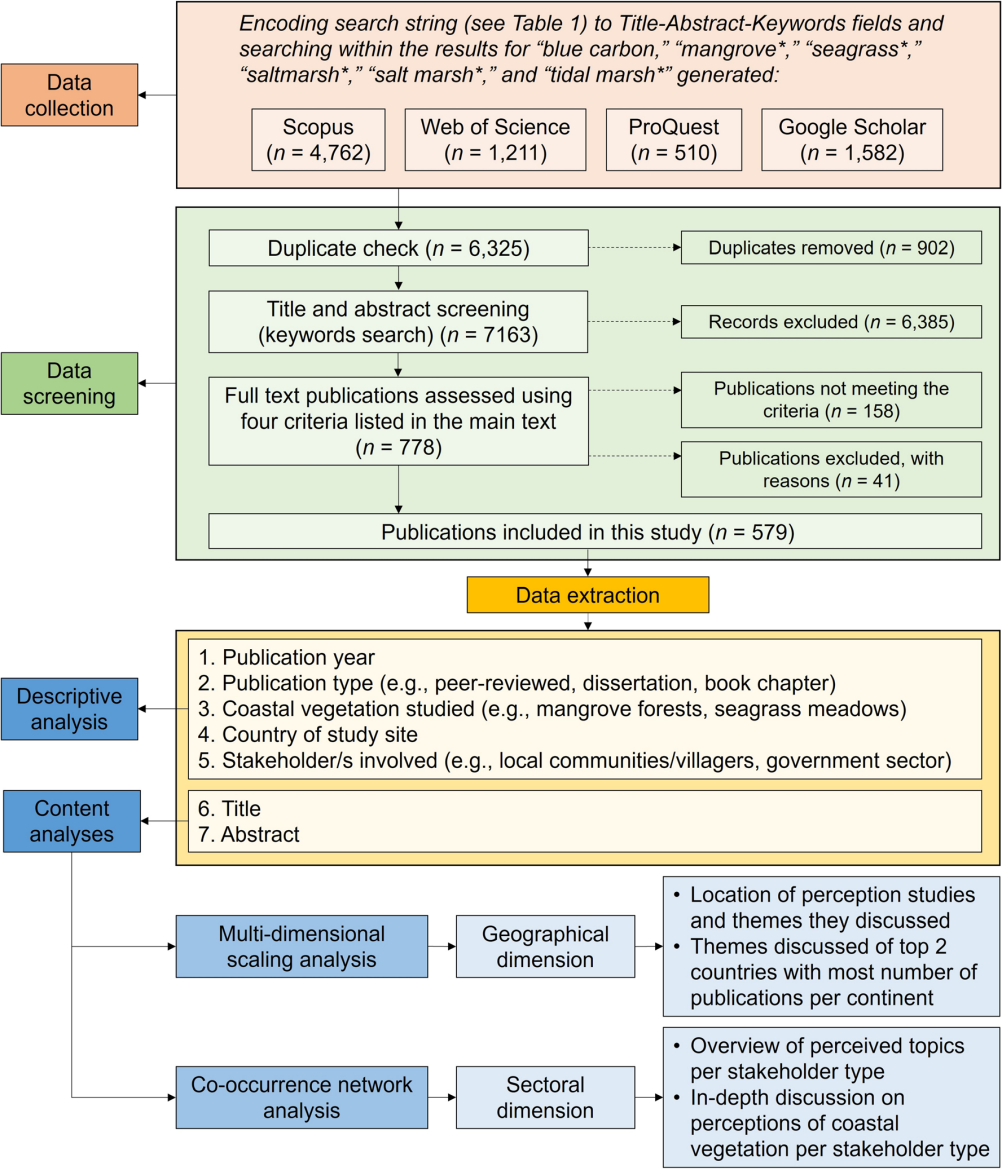
Schematic diagram of the methodological framework employed in this review. The data screening process was adapted from Quevedo et al. (2023)

Publications that potentially covered perceptions of coastal vegetated ecosystems were retrieved from four databases: Scopus, Web of Science, ProQuest, and Google Scholar (Fig. 1). These databases are commonly utilized in systematic reviews because they incorporate a range of publication types such as peer-reviewed articles, edited books and book chapters, dissertations/theses, and technical reports. The data collection process was conducted on January 8, 2024. There was no predefined cut-off date for the earliest articles considered in the search.

We developed a search string by combining insights from prior literature, discussions among authors, and commonly used terminologies in coastal and marine ecosystem research. To identify frequently used keywords, we consulted previous systematic reviews and meta-analyses on perceptions of coastal vegetated ecosystems (e.g., Bertram and Merk 2020; Jefferson et al. 2021). Additionally, we considered terminologies used in international environmental frameworks, such as the IUCN Habitat Classification Scheme and Ramsar Wetland Type Classification System, to ensure comprehensive coverage of relevant coastal vegetated ecosystems. Although we did not explicitly use online Thesaurus tools, we incorporated alternative spellings and variations (e.g., “saltmarsh*”, “salt marsh*”, and “tidal marsh*”) to enhance retrieval. Scholarly works on the definition of perceptions (e.g., Bennett 2016; Beyerl et al. 2016) were also used to identify similar terminologies. Applying this predefined search string (Table 1) to Title-Abstract-Keywords fields of the databases and searching within the results using keywords such as “blue carbon” OR “mangrove*” OR “seagrass*” OR “saltmarsh*” OR “salt marsh*” OR “tidal marsh*,” we identified a total of 8,065 potentially relevant documents.

**Table 1:** Search string applied in the data collection. An online database was utilized for Scopus, Web of Science, and ProQuest. The free application ‘Publish or Perish’ software (Harzing 2007) was used for Google Scholar

Search string	Database	Number of documents generated (*n)*
(“perception*” OR “perspective*” OR “perceiv*” OR “perceptive*” OR “observ*” OR “understand*” OR “interpret*” OR “evaluat*” OR “aware*” OR “value” OR “viewpoint*” OR “culture” OR “comprehend”) AND (“blue carbon” OR “mangrove*” OR “seagrass*” OR “salt marsh*” OR “saltmarsh*” OR “tidal marsh*” OR “coastal” OR “marine” OR “estuar*” OR “intertidal wetland*” OR “coastal wetland*”) AND (“local communit*” OR “local people*” OR “local stakeholder*” OR “local actor*” OR “coastal communit*” OR “Indigenous people*” OR “Indigenous communit*” OR “native people” OR “donor*” OR “government” OR “private sector” OR “business” OR “funder” OR “corporation” OR “NGO” OR “environmental organization” OR “non-profit”)	Scopus	4762
	Web of science	1211
	ProQuest	510
	Google scholar	1582

Data screening utilized the Rayyan platform, an online application for collaborative systematic reviews (Ouzzani et al. 2016). Rayyan assists reviewers in identifying relevant publications early in the title (Dos Reis et al. 2023) and abstract (Olofsson et al. 2017) screening stages. Following the Preferred Reporting Items for Systematic reviews and Meta-Analyses (PRISMA) protocol (Page et al. 2021), the initial step involved removing duplicates, resulting in the exclusion of 902 records (Fig. 1). Title and abstract screening utilized Rayyan’s features, highlighting the keywords used in our search (Table S1). Each title was reviewed, and, if deemed unclear, the abstract was screened. Subsequently, 6385 papers were excluded (Fig. 1). Reasons for exclusion included publications on topics such as coral reefs, remote sensing, ecological studies, and carbon stock assessments. The remaining 778 papers underwent a full eligibility assessment based on four main criteria: (1) written in the English language, (2) focused on at least one type of coastal vegetated ecosystem (mangrove forests, seagrass meadows, or tidal marshes), (3) explicit mention of the term “perception” and related terms (e.g., “perceive,” “perceptivity,” “viewpoint” [Jefferson et al. 2021]), and (4) presented perceptions data collected from a population (e.g., households, government leaders, business operators). The latter category could include, for instance, perceived benefits and values of the selected coastal vegetated ecosystems, perceived threats, or perceptions on the governance of these habitats.

After reviewing each publication, we excluded 158 papers that did not meet the inclusion criteria (Fig. 1). Among these, 29 papers were in languages other than English. We excluded non-English papers primarily due to practical constraints in translation and interpretation, which could introduce biases or inconsistencies into the analysis (Morisson et al. 2012). Systematic reviews commonly restrict language at the eligibility stage rather than the search phase to ensure consistency in data extraction and synthesis (Pieper and Puljak 2021). However, we acknowledge that excluding these papers may have led to the omission of regionally important insights, particularly from non-English-speaking coastal regions, which we discuss further in “Conclusions and future research opportunities” section. Additionally, we excluded 19 papers focused on the impacts of climate change and tourism on other coastal ecosystems, while another 41 papers were excluded for the following reasons: inaccessibility (*n* = 7), availability only as citations (*n* = 21) or abstracts (*n* = 8), or classification as non-primary data sources (*n* = 5).

### Data extraction and analysis methods

The 579 papers that met the inclusion criteria (Fig. 1) were organized by: (1) publication year, (2) publication type, (3) type of coastal vegetated ecosystem studied, (4) country of study site, (5) stakeholders involved, (6) title, and (7) abstract. Samples of data extraction are provided in Tables S1 and S2. The fifth category of stakeholders was further divided into public, private, and civil society sectors (Table S3). Stakeholders were categorized based on their primary role and function as described in the reviewed studies, following established governance and sectoral frameworks (e.g., Berkes 2010; Ostrom 2010; Bryson et al 2015). The public sector includes government agency representatives and village leaders, who play key roles in policy implementation, regulatory enforcement, and other forms of governance in coastal vegetated ecosystems. The private sector comprises individuals or companies engaged in market-driven activities, such as tourism-related groups, privately-owned businesses, and landowners. Individual fishers and farmers were classified under this sector due to their economic activities, such as harvesting coastal resources for income generation or operating for-profit aquaculture farms. The civil society sector includes community-based organizations, NGOs, and Indigenous people’s organizations involved in collective action, resource management, and advocacy. Fisherfolk and farmers’ associations were categorized under this sector when their role focused on governance, conservation or management rather than economic activities (Berkes 2010). This classification was applied based on the context in which stakeholders were described in each study. For example, if a study explicitly referred to fisherfolk associations as participants in mangrove forest management initiatives (e.g., Arumugam et al. 2021), they were categorized under civil society sector. However, if their role centered on economic livelihoods and resource extraction (e.g., Nyangoko et al. 2022), they were categorized under the private sector. A sample list of stakeholders and their sectoral categorization is provided in Table S2. Variables 1 to 5 were analyzed using descriptive statistics to derive frequencies and percentages using Microsoft Excel 2019 (version 1808).

We conducted content analyses to synthesize the studies of perceptions of mangrove forests, seagrass meadows, and tidal marshes by geography and sector (Fig. 1). For geographical distribution, we employed multidimensional scaling analysis in RStudio-based bibliometric package ‘Biblioshiny’ (Aria and Cuccurullo 2017). This algorithm maps high-dimensional data into lower-dimensional space, determining similarity or dissimilarity between pairs of objects and provides a basis for visualizing these relationships in two- or three-dimensional plots (Saeed et al. 2018; Iman et al. 2023). The method identifies clusters of related terms or documents, which is useful for understanding the overall structure and themes of the dataset (Iman et al. 2023). In our analysis, themes were generated based on titles and abstracts of selected publications. The themes helped with understanding the general research landscape on perceptions of coastal vegetated ecosystems within each geographical context. By accounting for the presence or absence of themes rather than simply quantifying their frequency, we mitigated potential bias arising from unequal research outputs and provided a more comprehensive assessment of the research landscape. We used VOSviewer 1.6.20 to perform co-occurrence networks analysis of words in the analysis of sectoral perceptions of the selected coastal vegetated ecosystems (Fig. 1) (van Eck and Waltman 2010). Co-occurrence networks, extracted titles, abstracts, or author-supplied keywords, can reveal research hotspots, general trends, and emerging topics (Chen et al. 2016; Catone et al. 2020).

## Results and discussion

### Data characteristics

**Fig. 2:**
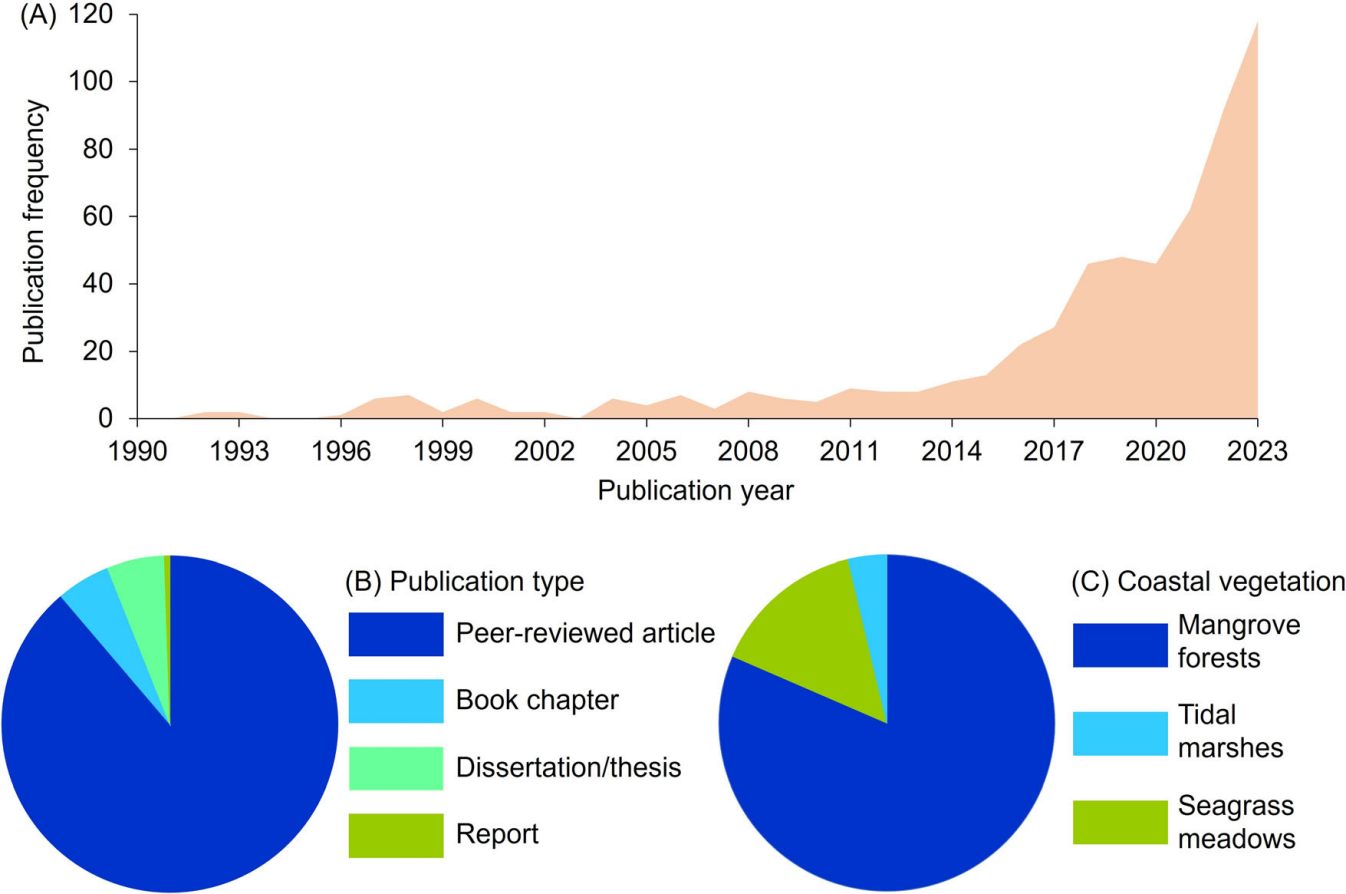
Overall characteristics of publications reviewed in this study. (**A**) Number of publications over time, (**B**) publication types, and (**C**) the type of coastal vegetated ecosystem studied

Our literature search identified a total of 579 publications, with the earliest two articles studying the perceptions of mangroves published in 1992 (Fig. 2A). These articles explored people’s understanding of the benefits of mangroves (Sukardjo and Yamada 1992) and growing awareness about the importance of sustainably utilizing mangrove resources (Ajiki and Miyagi 1992). Overall, the number of publications per year increased, from two in 1992 to 118 publications in 2023 (Fig. 2A). In total, there were 514 peer-reviewed articles, 30 book chapters, 32 dissertations/theses, and 3 reports (Fig. 2B).

Among the three types of coastal vegetated ecosystems examined in this review, studies on perceptions of mangrove forests dominated the selected publications (507 documents or 81%), whereas studies involving perceptions of seagrass meadows and tidal marshes were explored in 92 (15%) and 23 (4%) documents, respectively (Fig. 2C).

### Geographical distribution of perception studies

#### Global research landscape

The majority of publications (96%) were based on study sites located in tropical and subtropical regions, while the remaining 21 publications (4%) were in temperate zones (Fig. S1). Asia, comprising 48 countries across five regions, had the highest number of articles by continent (373 publications or 64%). Among these, the greatest number of studies were concentrated in Southeast Asia, especially Indonesia (182), the Philippines (46), and Malaysia (35). Africa accounted for 115 publications (20%), with Tanzania and Kenya represented in the most published studies (25 and 23, respectively). North and South America accounted for 34 (6%) and 22 publications (4%), respectively. Europe and Oceania were the basis for 18 documents (3%) and 17 (3%) of studies, respectively.

Trends observed in the global research landscape reflect the distribution of coastal vegetated ecosystems. For example, in terms of mangrove forest coverage, Asia accounts for the largest proportion, followed by Africa, South America, North America, and Oceania (Jia et al. 2023). In some cases, research output aligns with ecosystem coverage. Indonesia, for instance, has both the largest share of global mangrove forest coverage and the most published studies in our dataset (Fig. S1). However, this alignment is not universal. Countries with extensive mangrove ecosystems, such as Nigeria and Papua New Guinea, remain underrepresented in research, while those with smaller mangrove forest areas like the USA and China hosted a disproportionately high number of studies due to their well-established scientific infrastructure and relatively high levels of research funding (Jiang et al. 2022; Friess 2024).

It is important to note that trends observed in the social sciences, particularly research on perceptions of coastal vegetated ecosystems, diverge from those observed in ecology or biophysical sciences. Social science researchers often engage closely with local communities and government agencies, making the involvement of local collaborators essential to bridging cultural and institutional gaps. However, international collaborations between researchers from the Global South and Global North remain limited, which hinders the inclusiveness of context-specific research (Quevedo et al. 2023).

By focusing on perceptions of coastal vegetated ecosystems, our review aligns most closely with social science and policy research. Therefore, the distribution patterns in our dataset differ from those in other reviews that focus on ecological studies (Pang et al. 2024; Wang et al. 2024).

**Fig. 3:**
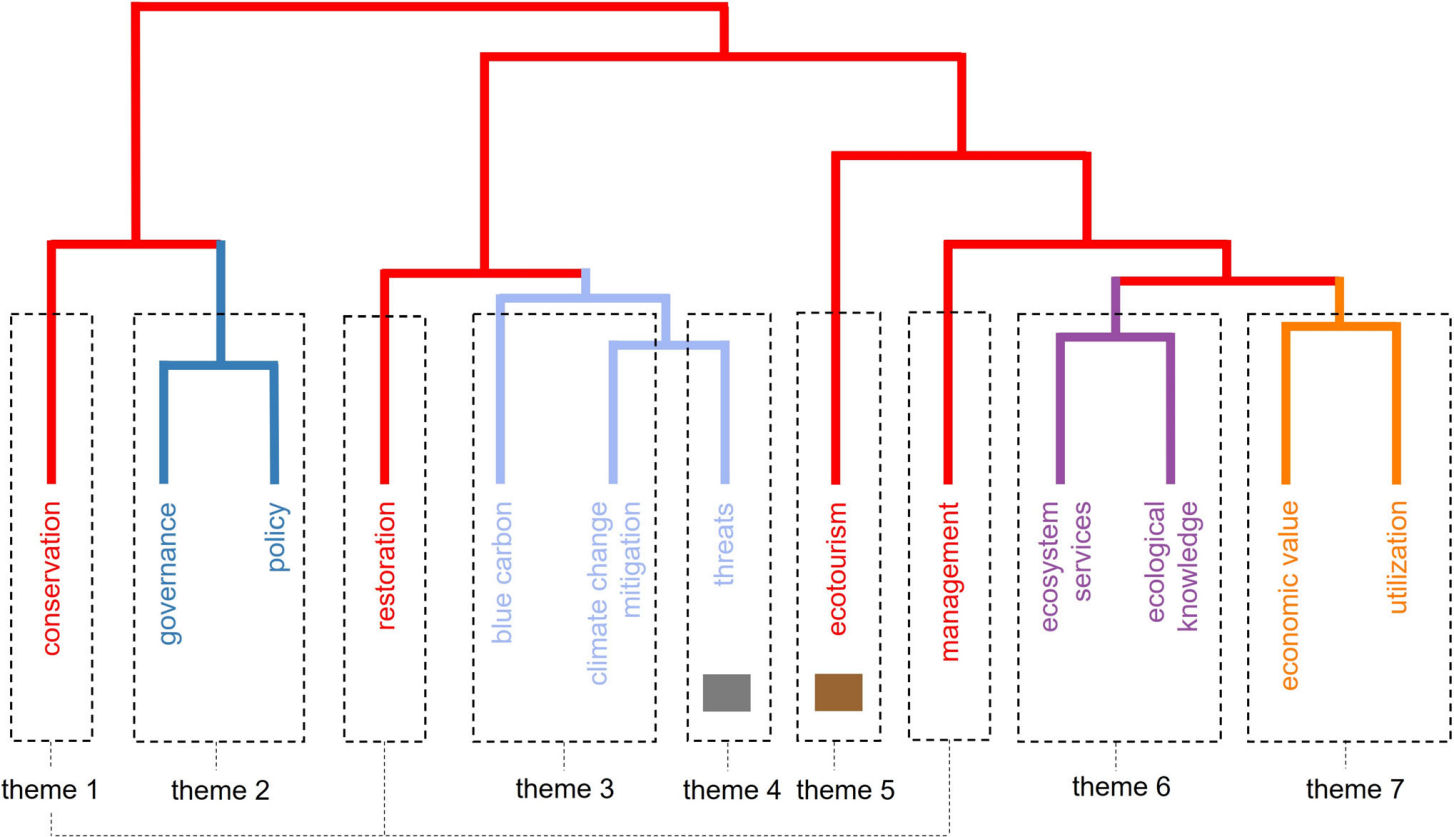
Multidimensional scaling-based dendrogram of title and abstracts and the seven thematic clusters

Country-level analysis of geographical trends afforded richer insights into general perceptions of coastal vegetated ecosystems on themes including management, governance, climate change mitigation, threats, ecosystem services, and utilization (Fig. 3). Studies that encompassed all seven themes were documented in 16 countries across six continents: (1) for Asia, Bangladesh, China, India, Indonesia, Malaysia, the Philippines, Sri Lanka, Thailand, and Vietnam; (2) for Africa, Gambia, Kenya, and Tanzania; (3) for Europe, Malta; (4) for North America, Mexico; (5) for South America, Brazil; and (6) for Oceania, Fiji (Fig. S1). In contrast, a study in the Solomon Islands of the perceptions of local people on access to ecosystem services provided by marine habitats (including mangrove forests), revealed only one theme (theme 6; Fig. S1) (Lapointe et al. 2020). By comparing urban and rural perceptions, Lapointe et al (2020) showed the impact of access to, and availability of, ecosystem services.

Our analysis also revealed that perceptions in different countries emphasized varying themes (Fig. S1), highlighting the unique socio-economic, environmental, and policy contexts shaping perceptions and priorities regarding coastal vegetation. For instance, perceptions on the management, conservation, and restoration of coastal vegetated ecosystems (theme 1) and the benefits they offer (theme 6) were the most prevalent themes, studied in 61 of 62 countries (Fig. S1). In contrast, articles that explored perceptions of ecotourism prospects (theme 5) of these habitats were recorded in 24 countries (Fig. S1). These articles mostly involved explorations of the ecotourism potential of mangrove forests in Asia (9 countries) and Africa (7 countries), where it has been perceived to improve mangrove forest management, increase awareness, and stimulate local economies (e.g., Huge et al. 2016; Runya et al. 2022).

Studies of stakeholders’ perceptions about the threats (theme 4) and economic value and utilization (theme 7) of services provided by coastal vegetated ecosystems are also well represented geographically, with themes 4 and 7 being the focus of studies in, respectively, 59 (95%) and 52 countries (84%). Notably, both themes are often explored together (84% of the countries; Fig. S1). For instance, perceptions of Indigenous people about unregulated and exploitative use of mangrove resources and monetary mechanisms to conserve and manage mangroves were studied in Australia and Fiji (Sangha et al. 2019). Community perceptions about major threats to mangroves and their impact on the sustainability of livelihoods were also investigated in Benin and Togo (Gnansounou et al. 2022).

Articles involving perceptions of the governance and policies related to coastal vegetated ecosystems were recorded in 43 countries (69%) (Fig. S1). Examples of these studies include investigating local communities’ perceptions about policy enforcement, institutional arrangements, and barriers to good environmental governance (e.g., Gayo 2022; Nijamdeen et al. 2023). Such explorations of perceptions have informed recognition that successful co-management of mangroves requires a combination of inclusive policy instruments and public participation through partnerships with government agencies (Begum et al. 2021).

Publications dealing with perceptions related to climate change mitigation benefits of coastal vegetated ecosystems collectively covered 36 countries (58%). Notably, several studies have evaluated public awareness of, and concern for, the carbon sequestration benefit of these habitats, which has been documented to be relatively low compared to other benefits such as food security and coastal protection (e.g., Quevedo et al. 2020; Lukman et al. 2021; Losciale et al. 2022). The impacts of coastal carbon offset projects (e.g., Sundarbans Mangrove Restoration project, Yagasu project) on people’s livelihoods have also been explored through the lenses of project developers’ and managers’ perceptions, showing both positive and negative outcomes (Herr et al. 2019). Mangrove forest restoration policies have further been investigated in relation to women, revealing gendered perceptions about the importance of a “long-lasting and shared governance” of these important ecosystems (Cormier-Salem 2017, p159). Perceptions about social and environmental justice (manifesting, for example, in concerns about the exclusion of marginal groups and the need for fairness in benefit-sharing) in regulating blue carbon projects (e.g., Mikoko Pamoja and Vanga Blue Forest projects) have been investigated by academics at the local level (Huxham et al. 2023).

#### Country-level research thematic trends

**Fig. 4:**
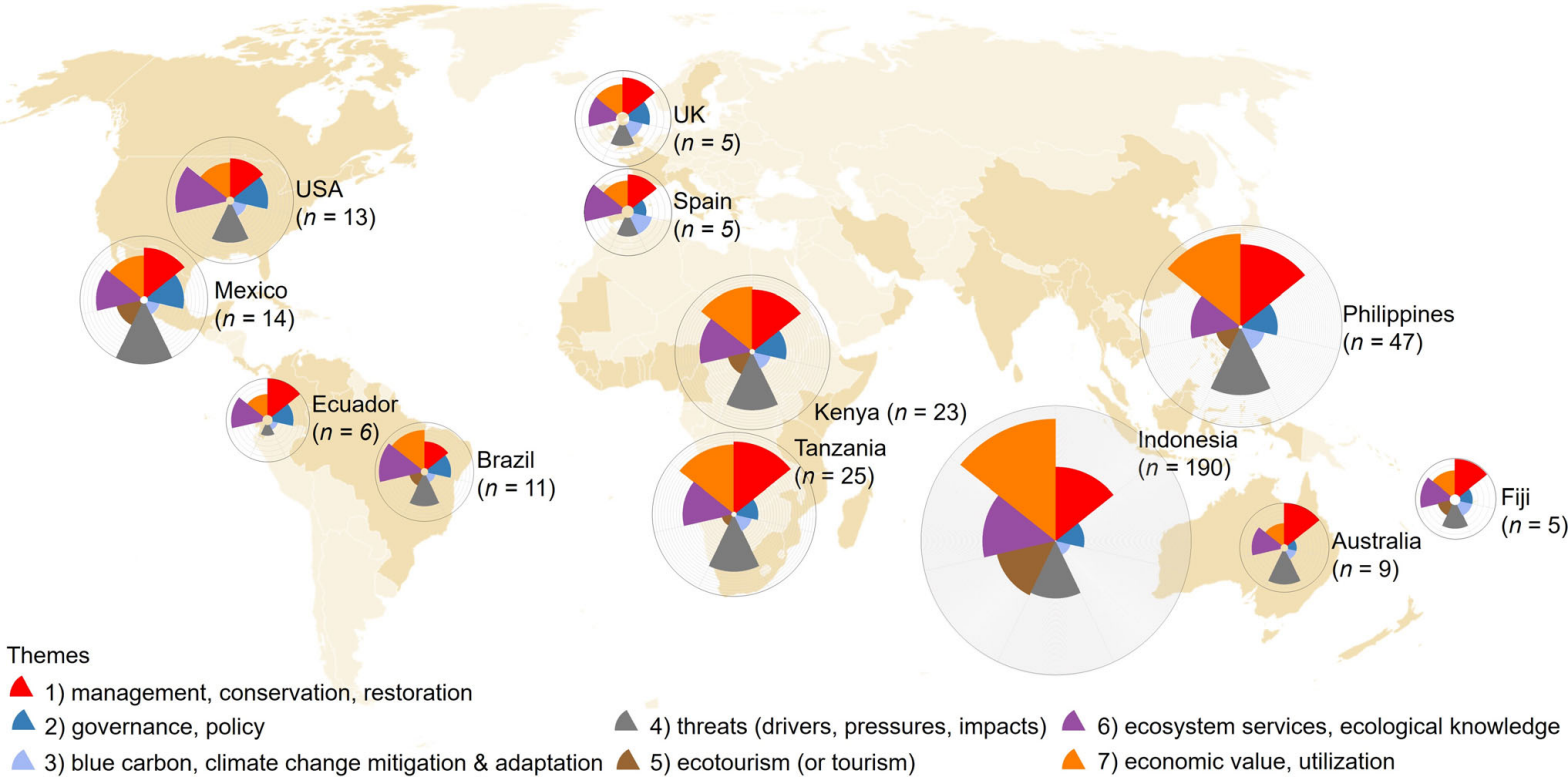
Top two countries with the highest number of publications per continent, illustrating the frequency of each theme studied

Thematic trends among groups of countries revealed some regional variations (Fig. 4). These underscore the diverse research priorities and contextual factors shaping perceptions of coastal vegetated ecosystems at the global scale. For instance, perceptions on the various utilizations of mangrove forests and seagrass meadows emerged as the most studied theme in countries such as Indonesia and the Philippines, comprising 168 (88%) and 43 publications (92%), respectively (Fig. 4). These two archipelagic countries support extensive coastal vegetated ecosystems (Himes-Cornell et al. 2018), with 60–70% of the populations of Indonesia and the Philippines living in coastal areas and relying on mangrove forests and seagrass meadows for their livelihoods and nutrition (D’Agnes et al. 2005; Stacey et al. 2019). Several studies emphasize stakeholders’ perceptions of mangroves and seagrasses in terms of their important contributions to livelihoods (e.g., fishing and gleaning of shells), local economies (e.g., tourism enterprise), health (e.g., medicinal herbs), and overall well-being (e.g., Damastuti and de Groot 2019; Kasim 2021; Nguyen et al. 2022). This same emphasis is observable in Tanzania and Kenya, where the perceived utilization and economic benefits of coastal vegetated ecosystems rank among the main foci, representing 21 (84%) and 19 documents (83%), respectively (Fig. 4). Of the estimated 60 million people living in coastal eastern Africa (including Mozambique and Somalia), many are reliant either directly or indirectly on the goods and services provided by two large marine biomes, including mangrove forests and seagrass meadows (UNEP-Nairobi Convention and WIOMSA 2015). These habitats support livelihood activities in eastern Africa, such as small-scale fisheries and tourism (UNEP-Nairobi Convention and WIOMSA 2015), with the economic value of seagrass meadows through its support for fisheries highlighted in Mozambique (Amone-Mabuto et al. 2023) and Tanzania (de la Torre-Castro and Rönnbäck 2004). In addition to these material benefits, one assessment of community-based ecotourism activities in mangrove forests in Kenya recognized the perceived importance of these habitats to cultural traditions and skills-based capacity building (Runya et al. 2022).

Studies investigating the perceived contributions of coastal vegetated ecosystems to conservation and sustainable management have been conducted in Brazil, Spain, and the USA (Fig. 4). In Brazil, perceived benefits of mangrove forests were identified relating to livelihood sustainability, personal satisfaction, mental and physical relaxation, and maintenance of traditional ecological knowledge (de Souza Queiroz et al. 2017). In Spain, historical and current social perceptions of the benefits of seagrass meadows were explored to identify the barriers to knowledge sharing and inform social communication plans (Baranano et al. 2022). In the USA, perceived protection benefits of tidal marshes were investigated against the backdrop of coastal hazard mitigation and climate change adaptation (Gray et al. 2017). These studies highlight the importance of recognizing the benefits and services of coastal vegetated ecosystems in supporting conservation and sustainable management measures (de Souza Queiroz et al. 2017; Gray et al. 2017).

Studies related to management perceptions of coastal vegetated ecosystems were explored in all of the publications identified in Australia (nine), Fiji (five), and Ecuador (six). In Australia, Losciale et al. (2022) surveyed the public to identify perceptions regarding the primary constraints to effective seagrass meadows management and restoration, emphasizing a lack of societal awareness about their benefits and importance to human well-being. In Fiji, interviews with representatives of Indigenous and local communities identified the value of incorporating traditional knowledge and cultural practices into management interventions in Indigenous and local communities (Pearson et al. 2019; Sangha et al. 2019). Felix and Hurtado (2019) reported a successful case of community-based management in Ecuador through the lens of fisher associations. That study found fishers who were given exclusive access to fishing areas became committed to protecting the mangrove forests and complying with local policies. In the UK, interviews with public (e.g., government), private (saltmarsh landowners and managers), and civil society (NGOs) sectors reported different challenges and benefits of livestock (e.g., cattle, sheep, horses) grazing on saltmarshes (McKinley et al. 2022). The interviews illustrated the diversity of values, personal connections, and sense of stewardship over saltmarshes, which highlighted the need for an integrated and multi-sector approach to management in properly accounting for these varying cultural, social, economic, and environmental values (McKinley et al. 2022).

In summary, our analysis highlights the global distribution of studies involving perceptions of coastal vegetated ecosystems and provides a general overview of the research landscape. We illustrate the diversity of themes across countries and regions in an overview of the range of actors involved in these studies, highlighting the role of multi-sectoral engagement in shaping perceptions of these habitats worldwide. In the next section, we further analyze how perceptions of coastal vegetated ecosystems by public, private, and civil society sectors may influence their governance.

### Sectoral perceptions of coastal vegetated ecosystems

The reviewed publications engaged a diverse range of stakeholders, encompassing public, private, and civil society sectors (Table 1). Notably, civil society is the best represented sector, with 458 (57%) publications. Within this sector, local community members (e.g., households, residents) constitute a significant portion, having been surveyed or interviewed in 236 (52%) of the studies. Other key stakeholders comprise NGOs, fishers’ associations, and other community-based organizations such as farmer’s associations, senior citizen’s associations, women’s associations, and Indigenous people’s organizations. The public sector’s involvement was identified in 30% (246) of the selected publications, with a majority (182 articles or 74%) investigating perceptions of representatives from various government agencies. Finally, the private sector accounted for 107 (13%) of publications. Stakeholders within this sector include tourism-related groups (e.g., boat operators, resort owners, and tour guides) and privately-owned businesses (e.g., fishpond owners, small-scale entrepreneurs), comprising 49 (46%) and 58 documents (54%), respectively.

**Fig. 5:**
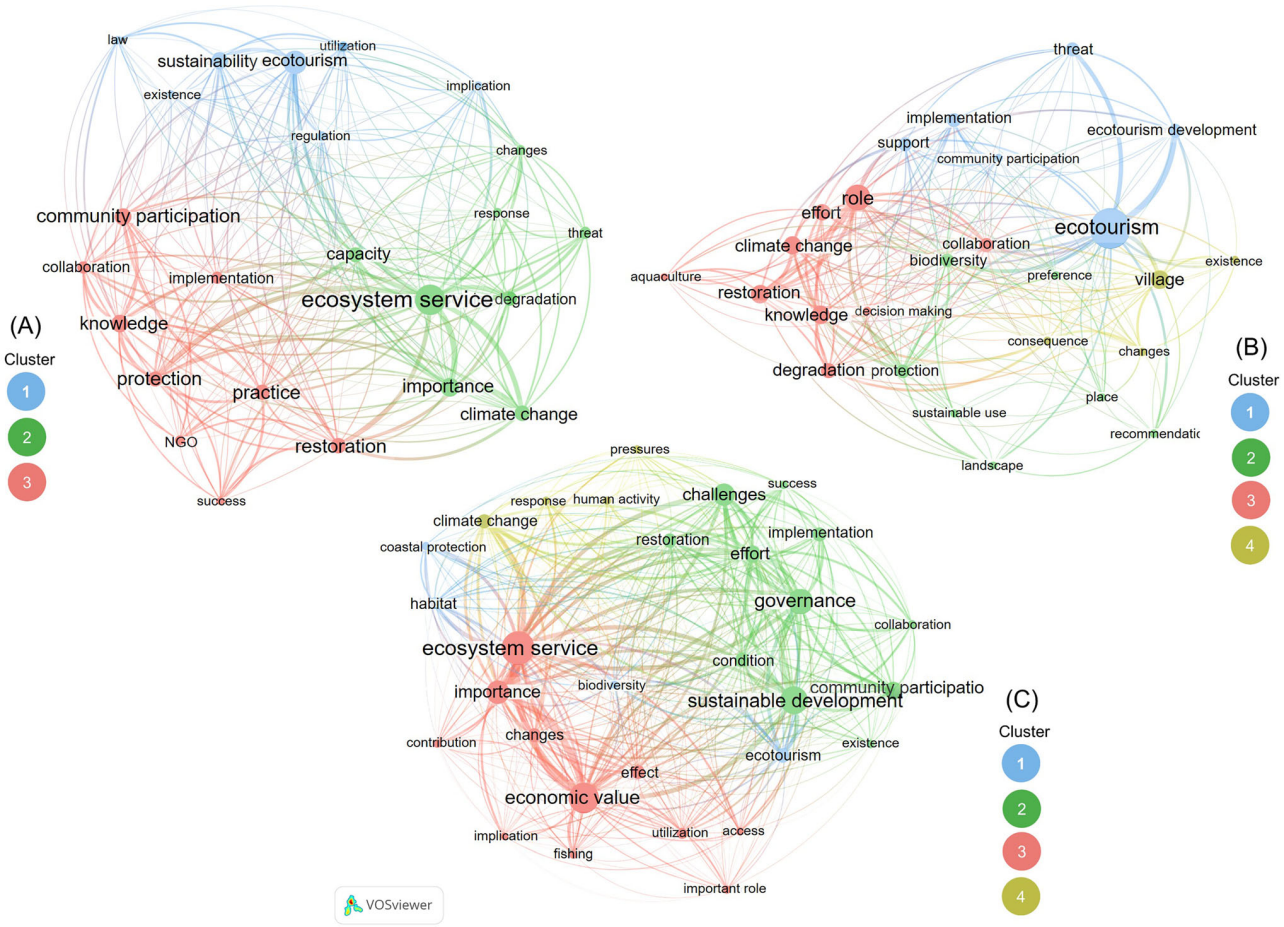
Co-occurrence network analysis of words from articles involving actors from the (**A**) public, (**B**) private, and (**C**) civil society sectors. Bubble (node) size indicates term frequency, while line (edge) thickness reflects the strength of cooccurrence

The word occurrence network provides an overview of the dominant themes and concepts in the literature involving sector-level perceptions of mangrove forests, seagrass meadows, and tidal marshes (Fig. 5A, 5B, 5C). Studies involving the public sector and civil society converge around the dominant theme of “ecosystem services”, highlighting a shared emphasis on the multifaceted (including monetized) benefits that these habitats provide (Fig. 5A, 5C). Articles mentioning the private sector further indicated a strong emphasis on the term “ecotourism”, invoking perceptions of economic opportunities and sustainable tourism development as crucial drivers for engaging with the selected coastal vegetated ecosystems (Fig. 5B). By delineating these sector-level perceptions, we can better understand the complexities of governance of coastal vegetated ecosystems and identify pathways for fostering collaborative approaches that integrate both ecological and economic considerations in their management. Results of the co-occurrence network were analyzed by sector in the following sections, where implications of these findings were also explored.

#### Public sector

Public sector perceptions of coastal vegetated ecosystems vary greatly, even within the same country. Governments are increasingly making decisions that tie economic and climate change mitigation valuations of these ecosystems to NDCs (Nationally Determined Contributions by country-level signatories to the 2015 Paris Agreement on Climate Change) (McHarg et al. 2022). Within and between government departments, however, many public sector representatives are unaware or unsure of the extent of coastal vegetated ecosystems within their jurisdictions and the range of options available to protect them (Dencer-Brown et al. 2022). Often, pro-economic development perceptions jostle against pro-conservation agendas to undermine the protection of coastal and marine ecosystems within NDCs (McHarg et al. 2022). In Indonesia, for instance, inter- and intra-governmental differences in perceptions of mangrove forests are reflected in the Ministry for Maritime Affairs (which has overseen Indonesia’s transition into Southeast Asia’s biggest aquaculture producer and is responsible for around half of the country’s mangrove loss) and the Ministry for the Environment, which encompasses competing agendas shaped by pro-mangrove timber and pro-conservation perceptions (Miller and Tonoto 2023).

While the term “public sector” is often associated with national government agencies, our review also considers perception studies involving local and provincial government officials, village councils, and other decentralized governance bodies engaged in coastal vegetated ecosystem management. The studies included in this review highlight that local government leaders and village representatives play a crucial role in shaping conservation and management strategies, often garnering greater trust among communities than higher-level government entities (Huxham et al. 2023; Low et al. 2024). Identifying public sector perceptions within and among governmental departments, as well as at the local level, could help elucidate similar conflicting issues and foster sustainable cooperation for the governance of coastal vegetated ecosystems (e.g., Thompson and Friess 2019; Quevedo et al. 2021).

In this review, articles mentioning public sector actors (Table 2) reflected a stronger emphasis on the term “ecosystem service” (Fig. 5A). The term frequently co-occurs with words such as “importance,” “degradation,” “climate change,” “restoration,” and “protection” (Fig. 5A). In part, this reflects a belief among some government representatives (including village and municipal heads) of the importance of environmental protection to maintain healthy coastal vegetated ecosystems and the benefits they generate (Thompson and Friess 2019; Quevedo et al. 2021). At the village level in particular, this perception underscores efforts to identify the causes of degradation and develop more effective ordinances and carbon sequestration projects (Quevedo et al. 2021). At the national level, there are case studies reporting the awareness of governments of contributions of mangrove forests and seagrass meadows restoration to carbon sequestration, although this is often complicated by an attendant lack of awareness of restoration techniques and insufficient funding (e.g., Mikely 2023; Mustapha 2023). These challenges are further exacerbated by issues within government institutions and departments. For instance, a network analysis highlighting Indonesia’s blue carbon governance process has reported that the government institution mandated with supporting the country’s climate commitments was not the central player in terms of climate engagements and public information (Ayostina et al. 2022). Their analysis has reflected other gaps among national-level institutions, including lack of inter-institutional coordination and mistrust.

**Table 2:** Sectoral categorization of stakeholders involved in selected publications (*n* = 579). *Percentage based on the total number of studies across all sectors (*n* = 811), **Percentage per stakeholder type per sector total; note that a publication could involve multiple sectors

	Frequency	Percentage
Public sector	246	30*
Government agency representatives	182	74**
Village heads/leaders	64	26**
Private sector	107	13*
Tourism-related groups	49	46**
Privately-owned business representatives	58	54**
Civil society sector	458	57*
Local communities	236	52**
Fisher associations	64	14**
Other community-based organizations (e.g., youth, religion-based, occupation-based)	63	14**
Non-government organizations	58	13**
Women’s associations	21	5**
Indigenous people’s organizations	16	3**

These challenges could be addressed through partnerships with other sectors, as discussed in the articles we reviewed. The co-occurrence network analysis also captured terms such as “collaboration,” “NGO,” and “community participation” (Fig. 5A), underscoring the need for multi- and cross-sectoral partnerships to overcome existing obstacles. For instance, some studies have reported that local government officials believe working collaboratively with environmental NGOs will help to safeguard their mangrove forest management projects and programs (Arumugam et al. 2021; Nyangoko et al. 2022). However, the public sector and NGOs can have different priorities and agendas. The former may prioritize adhering to regulations and legislation, whereas the latter may focus more on community empowerment or sustainable resource use, even if such local interests are not protected within existing laws and frameworks (Lin et al. 2015; Triyanti et al. 2017). Community participation plays a crucial role in the governance of mangrove forest management projects, not merely as a means of implementing government initiatives but as a collaborative process in which communities, local government bodies, and other relevant stakeholders share responsibilities (e.g., Arumugam et al. 2021; Ahmed et al. 2023). Rather than relying solely on government institutions, local communities actively contribute to planning and decision-making in environmental stewardship efforts. Additionally, collaborations across different levels of the public sector, including national agencies, municipal governments, and village leadership, is vital for effective management of coastal vegetated ecosystems. In the Philippines, for instance, village leaders and councilors recognize the importance of municipal government support in implementing conservation programs, though such support can be constrained by political and personal conflicts among local leaders (Quevedo et al. 2021). Furthermore, coordination between the municipal and central government agencies influences policy coherence, with studies highlighting the role of government officials in bridging gaps between national directives and local implementation (Arumugam et al. 2021). Recognizing the public sector’s multiple layers, from national ministries to village councils, is essential for understanding how governance structures shape community participation in conserving and sustainably developing coastal vegetated ecosystems.

#### Private sector

The private sector plays a complex and important role in the governance of coastal vegetated ecosystems, though its impact often mixed (Edbauer 2021). Private sector actors and institutions may be simultaneously involved in conservation and sustainable use initiatives while contributing to ecosystem degradation. In certain cases, the engagement by privately-owned businesses in conservation and restoration activities may be driven more by financial incentives or regulatory obligations than by actual environmental commitment (Edbauer 2021; Pham et al. 2022). Yet, private sector involvement in mangrove restoration and climate change mitigation—such as nursery management and tree planting—is increasing. The private sector plays a crucial role in supporting conservation efforts by funding projects, establishing environmental management systems, and engaging in conservation initiatives through corporate social and environmental responsibility (CSER) programs (Baker et al. 2020; Hattam et al. 2021; Nijamdeen et al. 2023).

In this review, we identified two sets of private sector actors: tourism-related (e.g., travel and tour agencies, resort owner, tourists) and farming and fisheries businesses (e.g., Joint Business Groups, fishpond owners, small-scale fish vendors) groups (Table 2). Ecotourism emerges as the dominant theme in the co-occurrence network (Fig. 5B), highlighting the complex spectrum of perceptions about its potential to contribute to conservation and create economic opportunities. The term frequently co-occurs with words such as “ecotourism development,” “threat,” “village,” “biodiversity,” and “collaboration,” illustrating the full spectrum of perceptions on ecotourism prospects in coastal vegetated ecosystems. These studies have documented perceptions ranging from valuations of growing intersections between ecological stewardship and business opportunities (e.g., Basyuni et al. 2018; Astikasari et al. 2023) through to the real and perceived negative impacts on society and the destruction of coastal vegetated ecosystems (e.g., Swangjang and Kornpiphat 2021; Zainal et al. 2023).

For example, ecotourism businesses recognize the vital role of mangrove forests in supporting local economies, such as through eco-friendly lodging and guided tours (Basyuni et al. 2018). In Indonesia, ecotourism has contributed to the preservation of mangrove forests while offering income opportunities to local communities through the sale of mangrove products and tour guiding services (Astikasari et al. 2023; Lukman et al. 2025). However, private sector involvement in ecotourism requires careful management to ensure long-term sustainability and to avoid unintended environmental impacts, such as biodiversity loss or land conversion for commercial purposes (Swangjang and Kornpiphat 2021; Runya et al. 2022). This is particularly critical in regions where businesses rely on healthy ecosystems for their operations (e.g., mangrove eco-parks, snorkeling sites). In Mexico, private investors, especially in the tourism industry, are driven by the need to ensure the sustainability of the ecosystems they depend on, aligning economic growth with environmental sustainability (Baker et al. 2020). Many in tourism sector are shifting from reactive to proactive environmental engagement as a way of safeguarding their resource base (Baker et al. 2020).

The second group of private sector actors identified in the reviewed studies includes businesses such as shrimp farming industries, major contributors to the destruction of mangrove forests. These industries are particularly reliance on coastal areas for pond construction and are responsible for significant mangrove deforestation (e.g., Hossain et al. 2013; Malik et al. 2017). Other private sector actors involved in mangrove ecosystem degradation include project developers, investors, and donors who fund or implement large-scale development initiatives that may inadvertently harm these critical ecosystems. While these actors play an important role in the economic development of coastal regions, their activities can lead to long-term environmental damage if not carefully managed (e.g., Edbauer 2021; Nyangoko et al. 2022). Their vested can thus lead to conflicts between governmental and private stakeholders, especially in decision-making over project priorities and trade-offs between environmental and economic sustainability (Hattam et al. 2021; Nijamdeen et al. 2024). For this reason, collaborations between the private sector, government institutions, and civil society should be well-communicated from the outset to ensure that diverse perceptions of value are integrated to maximize economic, conservation, and societal co-benefits. For example, cooperation might include providing permits to sustainably operate residential and production areas, while safeguarding adjacent mangrove ecosystems (Basyuni et al. 2018; Astikasari et al. 2023; Farid et al. 2023).

Understanding the motivations behind private sector engagement—whether driven by financial incentives, regulatory requirements, or a commitment to environmental stewardship—is needed to secure private sector participation in, and financing for the conservation of, coastal vegetated ecosystems. By gaining deeper insights into these motivations, we can refine governance models, improve regulatory structures, and develop more supportive legislative frameworks. This can help foster greater awareness and participation, particularly in cases where financial returns are not immediately evident (Baker et al. 2020; Edbauer 2021).

#### Civil society sector

Civil society includes the full spectrum of civilian-organized interests beyond the public and private sectors. In coastal vegetation scholarship, however, this sector is mainly mentioned in relation to local or coastal “communities”, and, to a considerably lesser extent, fishers’, farmers’, women’s and Indigenous groups (Table 2). This sector often represents the voices of individuals and communities most directly affected by the health of coastal vegetated ecosystems, emphasizing the practical implications of conservation measures, the need for equitable governance, and the direct economic and social impacts on livelihoods and well-being (Beyerl et al. 2016; Triyanti et al. 2017; Owuor et al. 2019). The literature on these habitats tends to assume “perceived ecosystem services and benefits to local communities”, underpinned by commonly held perceptions of mutual benefit in reciprocal human–nature relations (Come et al. 2023, p.1). This is also reflected in the co-occurrence network analysis, where the term “ecosystem service” is the most prevalent theme and frequently co-occurs with terms such as “economic value,” “coastal protection,” and “habitat” (Fig. 5C). For instance, in communities dependent on fisheries, the economic value derived from mangrove forests and seagrass meadows is integral to sustaining local livelihoods (Nyangoko et al. 2022; Amone-Mabuto et al. 2023). Similarly, coastal protection services provided by mangroves are highly valued by communities living in vulnerable coastal areas (Come et al. 2023). In contrast, local communities may not explicitly assign value to other benefits of coastal vegetated ecosystems such as their role in climate change mitigation (e.g., Lukman et al. 2021; Nguyen et al. 2022). Indirect ecosystem benefits may not be immediately visible or tangible to other stakeholders (e.g., Quevedo et al. 2020). The scientific complexities involved in understanding and communicating these less tangible benefits further complicates their place in perception studies (Twyman et al. 2015).

The civil society groups addressed in studies of coastal vegetated ecosystems often live in close proximity to them, and, as such, have clear views about their perceived threats and impacts. For example, in Tanzania, Mustapha (2023) investigated local community’s perceptions of the impacts of climate change, fishing activities and grazing by sea urchins on seagrass meadows and, consequently, their livelihoods. Similarly, in India, Chandra and Mukhopadhay (2022) examined the effects of poaching and illegal cutting of mangroves on crabs and fish yields, which are crucial for the economic sustenance of local communities, through the lens of women’s groups. In Indonesia, Sulaiman et al. (2023) surveyed members of the Bajo Tribe to understand mangrove degradation, which they attributed to extractive human activities such as illegal logging, mining, and conversion to agricultural lands. As end users who rely heavily on the ecosystem services provided by coastal vegetated ecosystems, these communities often possess inherited ecological knowledge and may thus be a trusted source of ideas relating to governance interventions needed for their effective management (Chandra and Mukhopadhay 2022; Mustapha 2023; Sulaiman et al. 2023).

While the public sector focuses on formal policy-making and regulatory mechanisms, governance in the context of civil society holds potential for a more inclusive and participatory form of management (Fig. 5C), which has been explored in several studies through the lens of fishers’, women’s groups and Indigenous communities (Owuor et al. 2019; Gevaña et al. 2021; Thoya et al. 2022; Senghor et al. 2023). Civil groups may “hold views that are necessary for policy change and improvement” (Owuor et al. 2019, p 172). Valuing and including their local knowledge in management strategies are thus important “to co-produce locally accepted solutions” (Senghor et al. 2023, p 1). However, this is not always the case, as perceptions of civil society groups are often shaped by their heavy reliance on private and public sector funding, which in turn influences their activities in coastal vegetated ecosystems. For instance, in Senegal, villagers support local participation in mangrove replanting but expect compensation for their efforts (Arumugan et al. 2021). Since civil society perceptions are not homogenous and vary widely across groups and individuals (Jefferson et al. 2021), conflicts within and between communities have been reported. Roy (2016) found that in Bangladesh, legal and illegal beneficiaries of mangrove ecosystem services (e.g., harvesters, residents) hold differing views on conservation, leading to failures in implementation.

## Conclusions and future research opportunities

This study synthesized 579 publications on perceptions of coastal vegetated ecosystems employing content analysis methods to provide insights into the general research landscape across: (i) geographical regions and (ii) sectors. Our results reveal a strong concentration of studies in tropical and subtropical regions, particularly in Asia. While a diverse range of themes were explored, the most prevalent focused on management, conservation, restoration, and ecosystem services. Perceptions of governance and carbon sequestration were less frequently investigated. Moreover, regional analysis highlighted distinct geographic differences; Asian countries often prioritize utilization and perceived economic benefits of coastal vegetated ecosystems while countries in North America and Europe tend to focus more on perceptions related to conservation and management.

In terms of sector-level perceptions, relevant literature predominantly focuses on civil society groups, with a strong focus on ecosystem services that are perceived to generate financial incentives, especially for livelihoods that attract tourists. Carbon sequestration functions tend to be featured less, likely because the benefits they yield are not immediately felt by local communities. However, at the national level, government institutions and civil society groups appear to be aware of the contributions of coastal vegetated ecosystems to climate change mitigation, but they may not always be able to act accordingly because of financial constraints and overlapping directives and policies (Ayostina et al. 2022; Low et al. 2024). For studies mentioning private sector actors and institutions, a prevailing emphasis on ecotourism perceptions is evident, recognizing a growing interest in funding both the livelihood and conservation functions of coastal vegetated ecosystems. For instance, in Indonesia and Malaysia, revenue from ecotourism has supported infrastructural development in mangrove-dependent communities and provided financial incentives for conservation and restoration initiatives (Blanton et al. 2024).

An increased number of studies of perceptions of our selected coastal vegetated ecosystems over the last three decades, evident in our results, suggests a growing interest in this topic. Our review builds on previous research to highlight the importance of understanding diverse stakeholder perceptions for more inclusive, sustainable, and effective coastal governance. Further research on perceptions of coastal ecosystems could be developed as follows:

1. Expand geographic coverage: Future studies should broaden their geographic scope, as the distribution of reviewed published studies is heavily skewed toward Asia, particularly Indonesia, the Philippines, and Malaysia. While this pattern may reflect the alignment between research output and the global distribution of coastal vegetated ecosystems, many countries in Asia and Africa with extensive coverage remain underrepresented (Ho and Mukul 2021; Friess 2025; Wang et al. 2024). This gap is likely due to differences in scientific infrastructure, research funding, and local engagement in research initiatives (Jiang et al. 2022; Quevedo et al. 2023; Friess 2025). Furthermore, geographic differences in culture, ecological conditions, and political and economic contexts make knowledge transfer to under- or unstudied locations challenging. Addressing this gap requires targeted research in relatively understudied locations, generating new insights on governance and comparing them to existing findings. Scholars could adopt successful methodologies from well-studied countries in Southeast Asia to uncover local perceptions, challenges, and opportunities related to coastal vegetated ecosystems. Although we did not analyze research methodologies in depth, our full-text assessment revealed that many studies employed similar approaches such as household surveys, focus group discussion, key informant interviews, as noted by Jefferson et al. (2021).
2. Address thematic gaps: Future research should address thematic gaps by increasing the focus on perceptions of carbon sequestration functions, carbon financing schemes, and carbon governance. Our review observed that while a majority of studies on “management” and other “ecosystem services” dominate the literature, research specifically exploring perceptions of “climate change mitigation” remains limited. Given the rising global interest in carbon markets and nature-based solutions, further exploration on how stakeholder perceptions of these conservations strategies is needed. Additionally, assessment of the economic and social trade-offs involved in such initiatives would provide useful insights.
3. Broaden the scope of stakeholder perceptions: Future studies should include a wider range of stakeholders involved in perception research. As emphasized in O’Leary et al. (2024) and Low et al. (2024), the long-term effectiveness of nature-based interventions into coastal ecosystems will require improved collaboration, coordination, and communication among different stakeholders across sectors. Future research could synthesize academic literature on the perceptions of public sector actors, such as national policymakers, regional environmental agencies, and intergovernmental bodies (e.g., Ruiz-Frau et al. 2019; Ayostina et al. 2022). Similarly, private sector perceptions remain underrepresented, with most studies focusing on tourism operators and small businesses. Further research could explore the perceptions of private investors, corporate entities engaged in carbon markets, and philanthropic organizations. Since most of the private sector’s role in conservation is documented in gray literature rather than peer-reviewed sources, incorporating industry reports, government publications, and NGO documents provide a more comprehensive understanding of business involvement in coastal vegetated ecosystem management. These sources can offer richer and more nuanced insights of the factors driving private sector engagement, as well as highlight challenges and opportunities not fully addressed in academic literature.
4. Emphasize seagrass meadows and tidal marshes: Greater emphasis should be placed on understanding perception of seagrass meadows and tidal marshes, which play vital roles in climate change mitigation, coastal protection, and biodiversity conservation. Investigating sectoral perceptions of these ecosystems could provide valuable insights into how they are valued and managed (McKinley et al. 2022). Future studies could also examine the social and economic factors influencing conservation efforts for these habitats, drawing comparisons with more extensively studied mangrove ecosystems.
5. Focus on actors in ecosystem degradation and conservation: Future research should explore the perceptions of actors directly involved in the degradation or conservation of coastal vegetated ecosystems, such as aquaculture developers, industrial investors, and government agencies responsible for land-use planning. Expanding the scope in this way could provide deeper insights into governance challenges, particularly in mitigating harmful practices, and inform potential strategies for more effective conservation.

## Supplementary Information

Below is the link to the electronic supplementary material.

Supplementary file1 (PDF 414 kb)
