# Supplementary material for: Perceptions of coastal vegetated ecosystems: A systematic review across geographical and sectoral dimensions
Source: Ambio. 2025 May 29;54(10):1563–80. doi: 10.1007/s13280-025-02193-x (PMC12405094; doi:10.1007/s13280-025-02193-x)
Supplement: Supplementary file 1 — Supplementary file1 (PDF 414 kb) [file 13280_2025_2193_MOESM1_ESM.pdf]

Perceptions of coastal vegetated ecosystems: A systematic review across geographical and sectoral dimensions

AUTHOR NAMES

Jay Mar D. Quevedo*[a], Michelle Ann Miller[a], Dixon T. Gevaña[b], Danny Marks[c], Daniel A. Friess[d], Prayoto Tonoto[e], David Taylor [f]

AFFILIATIONS

[a] Asia Research Institute, National University of Singapore, Singapore

[b] College of Forestry and Natural Resources, University of the Philippines, Los Baños, Philippines

[c] School of Law and Government, Dublin City University, Dublin, Ireland

[d] Department of Earth and Environmental Sciences, Tulane University, New Orleans, USA

[e] Riau Provincial Environment and Forestry Office, Pekanbaru, Indonesia

[f] Department of Geography, National University of Singapore, Singapore

CORRESPONDING AUTHOR ADDRESS AND EMAIL

*correspondence: Jay Mar D. Quevedo. Asia Research Institute, National University of Singapore, AS8, #07-01, 10 Kent Ridge Crescent, Singapore 119260. jquevedo@nus.edu.sg

Supplementary Table 1. Sample data extraction employed in this study.

Authors	Title	Abstract	Publication year	Publication type
Gill	Slag, steel and swamp: Perceptions of restoration of an urban coastal saltmarsh	A community group, in conjunction with local government and industry, has been working on aquatic and terrestrial restoration at a Wollongong saltmarsh, previously diminished in size and degraded by harbour reclamation and an urban rubbish tip. Students evaluate restoration progress to date and devise some interesting potential directions.	2005	peer-reviewed
Iftekhar and Takama	Perceptions of biodiversity, environmental services, and conservation of planted mangroves: A case study on Nijhum Dwip Island, Bangladesh	Restoration of mangroves is often considered a way to minimize losses incurred from their decline and to provide additional services to coastal communities. However, the success of restoration programs is often focused on biological or ecological criteria. The situation is no exception in Bangladesh, which houses the world's largest mangrove plantations. This study has been undertaken in a south-central estuarine island (Nijhum Dwip) of the Bangladesh coast and aims to understand societal perception on the achievements of a plantation program. Through 110 household interviews and seven group discussions, an assessment was conducted of peoples' perception about major flora and fauna of the mangrove ecosystem, benefits derived from the forest, present condition of the forest, causes of degradation, and ways to improve the situation. Around one-fourth of the respondents mentioned that they were highly dependent on the ecosystem. The most important perceived benefits were: provision of raw materials, prevention against natural disasters, climate regulation and soil retention. However, the majority (>80%) of the respondents perceived the ecosystem to be degrading. Encroachment and illicit felling were identified as the main causes of such degradation. In order to arrest the continued degradation allowed by conventional forest management flaws, adaptive co-management has been recommended to conserve this ecosystem in a more equitable way.	2008	peer-reviewed
Curado et al.	Public Perceptions and Uses of Natural and Restored Salt Marshes	Only a few restoration projects incorporate public perception in their monitoring. However, participation of local people is a key process if social benefits are to be achieved. This study analyses, through a survey, citizens' perceptions and usage of natural and restored coastal marshes in the city of Huelva (southwest Iberian Peninsula, Spain), as well as landscape preferences. Most of Huelva's citizens recognised the benefits of coastal marshes (75%), a perception which increased with level of education. The majority of the respondents showed a low level of knowledge about the eco-lo-gical services and functions of salt marshes. In addition, visitor numbers to the studied restoration project increased by 27% after restoration. Regarding landscape preferences, most of the respondents preferred the native Chenopodiaceae salt marsh, and the native Spartina maritima landscape was chosen less often. In light of our results, environmental	2014	peer-reviewed

		education campaigns should be carried out in the city of Huelva to increase its citizens' knowledge of salt marshes.		
Switzer	TAKING STOCK: Community perception of a mangrove restoration and alternative livelihood program in the Verde Island Passage, Philippines	Community-based management has a long history in the Philippines, especially when it comes to marine resources. The Verde Island Passage (VIP), located in the northern Philippines and dubbed the “center of the center” of the world’s marine biodiversity, is no exception. This case study looked at community perception of a mangrove protected area located in the VIP, in the small barangay (village) of Silonay, within the province of Oriental Mindoro. Using the Community Voice Method (CVM), this project sought to reveal the community’s perception of the current state of Conservation International’s mangrove restoration and alternative livelihood program established several years earlier. This study represents the first time CVM has been implemented specifically as a program evaluation tool.	2015	dissertation
Latif	Visitor perception on the potential of Matang Mangrove Forest Reserve for ecotourism	In peninsular Malaysia, Matang mangrove forest reserve is the larger mangrove area which is about 40,711 ha. Matang Mangrove Forest is a national treasure, it was lies under federal and state authorities. Since early 1904, Matang Mangrove Forest being forest reserve with systematic management. This study was conducted at Matang mangrove forest to identify the potential of Matang mangrove forest as an ecotourism destination. There 137 questionnaire was distributed to the visitor during weekend and public holidays in the month of Feb 2016. This study finds that there are three significant factors that influence potential of Matanf Mangrove by visitor perception. The factor are satisfaction and need, relaxation and leisure and Malaysian certificate of educational. The research finding gives an understanding on what people's needs and wants for their recreational activities where it is useful to the related agency for their future management purpose.	2016	dissertation
Queiroz et al.	Neglected ecosystem services: Highlighting the socio-cultural perception of mangroves in decision-making processes	Despite the increasing recognition of the need to conserve mangroves, degradation has continued during the last two decades due to ineffective and non-inclusive decision-making processes exclusively based on economic factors. The purpose of the present study is to give tools to mangrove conservation management and policy, exploring the sociocultural valuation of the ecosystem services of mangroves through a case study in northeastern Brazil, an area highly impacted by shrimp aquaculture. We used a mix of methods to complement ecosystem services identified in the academic literature with those perceived as such by local people. We analyzed these locally perceived mangrove services in relation to community livelihoods, and highlighted that local people identified four additional cultural services related to maintenance of Traditional Ecological Knowledge (TEK), creation and maintenance of social relationship, personal satisfaction and mental and physical relaxation. This demonstrates that local people have a symbolic relationship with the mangrove forest, which goes beyond the material approach normally used to evaluate ecosystem services. Such findings suggest that the socio-cultural dimension of mangrove services needs to be considered by policy-makers	2017	peer-reviewed

		as an indispensable criterion for confronting the key challenges in coastal ecosystems conservation		
Marican et al.	Public perception towards sustainable mangrove forest programs in Malaysia	The mangrove forest has been an important vegetative aspect in maintaining an ecosystem balance. Among its benefits is to cover the source of timber, charcoal, woods, herbs, and fish and shrimp culture where all of this can be valued as economic resources. Even with vast benefits it offers, the number of mangrove forests are constantly declining and if there are no actions being taken, the mangrove forests may face the possibility of extinction. Degradation of mangrove forests in Malaysia is mainly due to development and agriculture. A sustainable mangrove forest is not something that is newly introduced in Malaysia as the Matang forest is known for its sustainable forest management for over 100 years. Sustainable mangrove forest programs do not only involve the necessary authorities but also the public as their participation in replanting and managing the mangrove forest. Thus, this study aims to determine the public perception towards sustainable mangrove forest programs in Malaysia. Data were collected via face-to-face interviews with respondents from the states which have high percentage of mangrove forests such as Perak, Sabah, Selangor, Johor, and Pahang. Prior to actual data collection, a pilot study was conducted in Kuala Selangor with 100 respondents. Respondents were selected by using a simple random sampling technique from mangrove and non-mangrove areas with a total of 871 respondents. Data collected were analyzed using descriptive analysis and chi-square analysis. Descriptive analysis has found that the public perception towards the importance of mangrove forest is high with the overall mean score of 4.09. The chi-square analysis revealed that gender, age, education level, income, marital status, and family members have significant relationships with the public perception towards sustainable mangrove forest programs. The insight of this study can help the government and NGOs to organize campaigns and programs related to sustainable mangroves forest more effectively.	2018	peer-reviewed
Elggren	People's perception of seagrass ecosystems-a Step Zero analysis in establishing marine protection of seagrass around Gotland, Sweden	The marine environment is under stress. This study will address seagrass around the island of Gotland, Sweden. Seagrass is a marine flowering plant which grows on the seabed and provide a multitude of important ecosystem services such as nutrient cycling, carbon sequestration, erosion mitigation and serve as habitats for primary fisheries. Due to the many benefits it provides seagrass is an important species and needs protection. Studies show there is a considerable lack of knowledge and indifference from the general public regarding the ecosystem services of seagrass meadows. Such indifference threatens seagrass meadows and their important ecosystem services. A survey made in this study indicates respondents are slightly unsure when distinguishing seagrass from other underwater fauna and applying features to it. However, a certainty exists that the Sea is important, and we should take care of it. Likewise, there is an awareness of the environment around Gotland and that is has deteriorated some over time, responses mostly reflected on fish catches, sea wrack and water clarity. Further, respondents are	2019	dissertation

		not always fully aware of how humans already benefit from ESS provided by seagrass. Consequently, the results indicate an uncertainty in the understanding between daily human activities (eg boating, fishing, handling of sea wrack) and potential changes of ESS. After answering the survey many expressed a curiosity and concern about seagrass but simultaneously felt unsure of how to protect or limit the destruction of seagrass. This indicates there is a possibility to inform, include and engage the general public as a stakeholder group. In the Baltic Sea the situation is severe due to eutrophication, toxins and overfishing causing certain ecosystems to collapse and the seabed becoming oxygen deficient. The Baltic Sea is under pressure, international and national environmental policies promote a higher protection and mitigation of stressors advocating an adaptive natural resource management and sustainable development. This thesis investigates what people's perception is about seagrass and the ecosystem services it provides around Gotland. It further looks at the process of implementing environmental protection and conservation and how this is dependent on active participation from the general public. The research aims to conduct a Step Zero analysis, establish a basis of stakeholders' perception of seagrass as a resource. The analysis is a pre-test and can provide a foundation of how to optimize and proceed in a potential deepening of marine protection. It further explores in what way information can be communicated to help illustrate the benefits and necessity of natural resource management. Using empirical data from a survey circulated on Gotland, this thesis explores what information is relevant for the local community to increase the awareness seagrass and the ecosystem services seagrass benefit provides. By determining if and how people perceive different aquatic benefits and threats, natural resource management may provide information and participation measures that can lead to a gradual alteration of behavior.		
Vegh, and Potouroglou	High Level Assessment of Seagrass Ecosystem Services in West Africa-Perception of Stakeholders	Seagrasses provide valuable ecosystem services–benefits to humans–but are now being lost globally at rapid rates due mainly to anthropogenic stressors. Ecosystem services are defined as the benefits that humans derive from the environment, in this case, from seagrass ecosystems. This report provides an initial assessment of seagrass ecosystem services in seven countries of West Africa, including Mauritania, Senegal, The Gambia, Guinea Bissau, Guinea, Cape Verde and Sierra Leone. Many of the ecosystem services that seagrass ecosystems outside of West Africa provide have values estimated over five degrees of magnitude, between USD 11 and USD 2.4 million per hectare. Only two values were published on West African seagrass species USD 136 and USD 1,226 per hectare per year, for nursery habitat and fish habitat, respectively. Based on regional opinion of experts, seagrass ecosystems are widely recognized as providing valuable benefits to the local, regional, and global community. In order of perceived importance, ideal considerations for further research, the top five are: (1) biodiversity habitat, (2) fish and nursery habitat, (3) sediment stabilization, (4) climate change mitigation through carbon storage, and (5) water quality regulation. The importance of seagrass ecosystem	2019	report

		services is widely shared among regional experts. Experts recognize that local communities depend on seagrasses to a noticeable extent, and that seagrasses are threatened by multiple stressors, both anthropogenic and natural. The importance of ecosystem services related to culture, art, and design, a sense of place, and general societal health and well-being have been revealed. Regional experts showed an understanding of the threats seagrasses face, which as a group they listed in order of importance as (1) human disturbance and development, (2) pollution, (3) fishing related threats, (4) climate change, and (5) lack of information. There is a general understanding of ineffective and poor management of seagrasses in West Africa, driven mostly by lack of financial resources and priorities in policy and legislation. Further research should prioritize updated seagrass maps, a complete list of seagrass ecosystem services, including social services, primary economic valuation studies, and surveys or interviews with beneficiaries that benefit from seagrass ecosystem services. Economic values of ecosystem services need to be better incorporated into policy decisions, but values that currently exist in the literature should be treated with caution.		
Nessa et al.	The role of women in the utilization of Enhalus acoroides: livelihoods, food security, impacts and implications for coastal area management	Coastal and island communities depend heavily on marine resources for their food and income. During the west monsoon (September to February), fishermen rarely go to sea, and their activities are limited to the small-scale fisheries, including gleaning the Enhalus acoroides fruits. Participants in this small-scale fishery sector are mainly women; with little or no skill required, these gleaners are often overlooked by the district level fisheries-related census. This case study focused on the role of coastal women with different levels of knowledge and utilization of E. acoroides in three cities/districts of South Sulawesi. This study aimed to determine the contribution of E. acoroides fruit collection to coastal community livelihoods and food security, to identify the impacts, and to seek alternative solutions for seagrass management. Qualitative methods were used to gather information on E. acoroides fruit collection, fishery knowledge, livelihood contributions, perceptions of seagrass condition, and alternative management solutions. Around 90% of E. acoroides fruit collectors were women aged between 30 to 80 years old. Women contribute greatly to livelihoods and food security in coastal areas because they play dominant roles in household affairs and the sale of fishery products. Unfortunately, the methods currently used for collecting E. acoroides fruit can be destructive and may have contributed to the decline in seagrass cover. However, prohibiting the collection of E. acoroides would not be an appropriate solution. A win-win solution is needed to empower women while simultaneously managing seagrass ecosystems sustainably.	2020	peer-reviewed
Quevedo et al.	Perceptions of local communities on mangrove forests, their services and	The mangrove ecosystems which provide diverse benefits to local communities are vulnerable to natural and human-induced threats. Existing policies and decision-makers in the Philippines are gearing towards the integration of physical, ecological, and social elements in managing these ecosystems. To date, the linkage between the policies and	2020	peer-reviewed

	management: implications for Eco-DRR and blue carbon management for Eastern Samar, Philippines	the direct beneficiaries (i.e. coastal communities) of the ecosystem services is, however, largely unexplored from local perspectives applying quantitative methodology. Thus, we conducted household surveys to the coastal villagers in Eastern Samar. By doing so, we provide basic information for the scientists and policymakers on the following elements of one the blue carbon ecosystems, mangroves; (1) resource utilization, (2) level of awareness on ecosystem services and existing management plans, and (3) perceptions on natural and anthropogenic threats. Results of the survey show that the utilization of mangrove ecosystem services is influenced by social demography and level of awareness of the locals. The trends of the locals' utilization and perceptions on the diverse ecosystem services may provide evidence for their active involvement in protecting these resources. To enable more holistic and sustainable management, this study suggests the value of including coastal communities in contextualizing management plans, particularly for the areas often visited by natural hazards.		
Sadono et al.	Local indigenous strategy to rehabilitate and conserve mangrove ecosystem in the southeastern gulf of kupang, east nusa tenggara, Indonesia	The existence of local communities around mangrove ecosystems plays essential role to support the effort of conservation programs in this area. This study is aimed to investigate a set of situation faced by local communities in the southeastern Gulf of Kupang (SGK), East Nusa Tenggara Province which led to the rehabilitation of once degraded mangrove forests in SGK and the strategies to conserve the recovered mangrove forests. A case study approach was developed using purposive sampling to collect information regarding the historical situation of mangrove forests in SGK. Further, remote sensing method using multi-temporal observation data was used to investigate the changes in mangrove cover from 1994 to 2019. This study revealed that a series of situations became the fundamental of the success in retaining the existence of mangrove ecosystems in SGK. First, the negative impacts of mangrove degradation affected the communities badly in relation to their livelihoods in fisheries and marine sector as well as other environmental services. Then, this situation led to the emergent of a local champion to initiate mangrove rehabilitation efforts which firstly did not get attention from most of the communities. After some initial successes, the efforts of the local champion was then followed by other members of communities, triggering a bigger scale of mangrove rehabilitation. Having the mangrove recovered, the communities set of highly strict local indigenous rules in which every indigenous people who conducting illegal logging in the mangroves would be expelled from the village, while a large fine was given for outside people who did the similar action. Currently, more than 90% of respondents have understood the benefits of mangroves and derived advantages from it, particularly in improving their prosperity and security. The results of the success of mangrove rehabilitation and conservation in SGK was confirmed by the increasing extent of mangrove vegetation using remote sensing data. The case study of rehabilitation and conservation in SGK provided valuable learning for communities in other areas.	2020	peer-reviewed

Firdaus et al.	Mangrove Forest Restoration by Fisheries Communities in Lampung Bay: A study based on perceptions, willingness to pay, and management strategy	Mangroves provide benefits and various services to local communities living along coastal areas, particularly fishery communities. Fishery community perceptions are significant in determining attitudes towards improving mangrove conditions, which can also be addressed through restoration activities. This research was conducted to analyze fisheries communities perceptions, willingness to pay (WTP) for mangroves restoration, and mangrove forest management strategies. Field surveys were conducted from July-August 2019 and February-March 2020. Primary data were collected from respondents in four regions (Kalianda Regency, South Lampung Regency, Bandar Lampung City, and Pesawaran Regency) in Lampung Province, Indonesia, which consist of fishers, shrimp farmers, crab and wood seekers, and finfish farmers. The respondents were 193 people, and four experts were involved in the policy scenario analysis. Results revealed a gap in the value of WTP among fishery community groups, in which the average value for fishers is lower than shrimp farmers. The years of formal education significantly influenced the WTP for mangrove restoration. Based on the scenario analysis, scenario 01 become a priority strategy, where four policies (P1 = Mangrove ecotourism development in Lampung Bay; P2 = Mangrove knowledge education and training on processing mangrove based products; P3 = Restoration and conservation of mangrove forests; and P4 = Community-based management for mangrove forests utilization) show high likelihoods to be simultaneously implemented for mangroves management, with mangrove ecotourism policy as the most decisive policy. For future research, other explanatory variables can be added, such as information on family member characteristics, and to develop a bottom-up policy scenario by identifying and involving the role of the local community.	2021	peer-reviewed
Gayo	Local community perception on the State Governance of mangroves in Western Indian coast of Kinondoni and Bagamoyo, Tanzania	Sustainable conservation of mangrove forests has been constrained by marginalising some key stakeholders in resource governance in Tanzania. The present study assessed the perceptions of local communities towards the state management of mangroves on the western Indian coast of Kinondoni and Bagamoyo. The mixed method design was used to collect data from 306 respondents from Kaole, Mlingotin, Pwani-Kunduchi, and Mtongani villages via household questionnaire surveys, interviews with key informants, focus group discussions, and documentary reviews. The overall mean score on a 5-point scale for respondents in both study sites was 3, indicating neutral perception. The perception of respondents towards state management of mangroves for ecological benefits was relatively higher (M = 3.92, SD = 0.18) than for socioeconomic context (M = 3.02, SD = 1.03), t (128) = 4.7, p < 0.01, d = 0.51) in Kinondoni. The same results were revealed in Bagamoyo as higher perception for ecological gain (M = 3.43, SD = 0.67) than for economic benefits (M = 2.63, SD = 0.86), t (133) = 5.3, p < 0.01, d = 0.63. Multiple regression models revealed that local community perceptions of state management of mangrove forests in both study areas can be explained by education level (Î² = 0.168, p = 0.012), knowledge of reserve rules (Î² = 0.187, p = 0.013),	2022	peer-reviewed

		household distance from reserve boundary (Î² = 0.798, p = 0.003), and occupation (Î² = ?0.162, p = 0.001). The study revealed that respondents were satisfied with the state's management of mangroves for ecological gain, but they were not supportive of management when economic issues were discussed. The study recommends the state management of mangroves to become inclusive and recognize the neighboring communitiesâ€™ entitlements; an adequate contribution of revenue from mangrove protection to local livelihood; and the provision of environmental education to communities on the potential of ecosystem services to increase their social acceptability.		
Nyangoko et al.	Local perceptions of changes in mangrove ecosystem services and their implications for livelihoods and management in the Rufiji Delta, Tanzania	Understanding the status and trends of ecosystem services (ES) in a changing environment is important for identifying effective management measures of multifunctional mangrove ecosystems. Mangroves and their ES are jeopardized by a complex set of factors, with impacts that are experienced at local levels, especially in developing countries, where people often rely directly on natural capital for their livelihoods and well-being. This study was set to explore how local communities in the Rufiji Delta, situated in central coastal Tanzania, perceived the status and trends in mangrove ecosystem services (MES), associated drivers of change and the impacts of changes in MES on local livelihoods. A mixed methodological framework (including focus group discussions, key informant interviews, household surveys and direct observations) was used. People from villages close to mangroves rated the status of MES higher than those in villages distant from mangroves. Provisioning services (P) were often perceived to be in a worse and more declining state than regulating (R), cultural (C) and supporting services (S). A decrease in availability of poles and firewood (P), decline of fish habitats (S) and an increase in education value (C) were the most commonly perceived changes of MES in the study area. Illegal harvesting of mangrove poles, rice cultivation, climate change and inadequate management were seen as the most critical drivers of mangrove degradation, although the perceptions differed between sites. Rice farming was perceived to be a primary cause of mangrove loss by communities far from mangrove forests, while illegal exploitation was identified as the major driver by communities near mangroves. Fishing, collection of poles and honey were perceived as the most impacted livelihoodsdepending on MES. This together with the comparatively low status and declining trend of these MES indicate that they should be of high management priority as indicated by the first order management index used in this study.	2022	peer-reviewed
Amone-Mabuto et al.	Coastal community's perceptions on the role of seagrass ecosystems for coastal protection and implications for management	Seagrass meadows provide important ecosystem services to coastal communities, which include ecosystem-based adaptations for coastal protection; however, globally seagrasses are among the least conserved marine ecosystems. In order to develop and provide accurate and efficient decision-making processes and conservation actions, an understanding of the importance of ecosystem services for people's livelihoods and wellbeing is key. Using social research methods, such as Nominal Group Technique,	2023	peer-reviewed

		Key Informant Interviews and household surveys, this study explored the association between seagrass ecosystem services and the perceptions of coastal communities on socio-ecological values including ecosystem-based adaptations in Maputo and Inhambane Bays, Mozambique. Fishermen identified support to fisheries production as crucial services provided by seagrasses to coastal communities. Respondents (93%) perceived that coastal erosion is occurring in the region and that it has accelerated in the last 20 years due to more frequent floods following cyclones. In addition, coastal development (34.9%) together with destructive fishing practices (31.3%) were stated as the main anthropogenic causes of coastal erosion as seagrasses diminish. The coastal communities (76.3%) proposed marine ecosystem restoration as the first measure to prevent coastal erosion. The coastal respondents also identified seagrass regulatory ecosystem services being central for their social and economic wellbeing and demonstrated (92%) a desire to participate in seagrass restoration in their region. This study found a high level of awareness by local communities concerning the values and socio-ecological function of seagrass meadows. This provides a strong foundation to develop effective conservation and management measures in order to ensure sustainable solutions to build resilience and stronger ecosystem-based adaptations against climate change.		
Nijamdeen et al.	Delineating expert mangrove stakeholder perceptions and attitudes towards mangrove management in Sri Lanka using Q methodology	Effective decision-making is key to the successful conservation and management of natural resources. Mangrove ecosystems all over the world provide an array of ecosystem goods and services and are managed by a wide range of stakeholders representing various sectors. The position of mangroves in the land-sea interface and the rapid development of coastal areas in the Global South make mangrove conservation and management more challenging than the management of other coastal ecosystems. Sri Lankan mangroves are degrading due to numerous natural and anthropogenic causes in recent years. Mangrove degradation in Sri Lanka is further exacerbated by the economic crisis following the COVID-19 pandemic. The coastal communities near mangroves in Sri Lanka heavily depend on mangrove goods and services (more than before, due to a lack of livelihood alternatives), despite formal no entry rules by the government. This study's objectives are to delineate the viewpoints of mangrove management experts to understand current mangrove management in Sri Lanka and to provide baseline data for effective decision-making. We used Q methodology, during which 71 mangrove experts representing 21 stakeholder groups were asked to individually rank statements regarding mangrove management. These rankings were subsequently clustered using Principal Component Analysis, allowing the identification of clusters of opinions regarding mangrove management. Stakeholder's perceptions were clustered into three discourses: community-oriented management, government-oriented management, and management in synergy between government and communities for effective mangrove conservation. Our findings emphasize the multifaceted nature of	2023	peer-reviewed

		mangrove management in Sri Lanka, revealing diverse perspectives among stakeholders. Our results further highlight the need for a collaborative approach to the co-management of mangroves in Sri Lanka. We recommend that mangroves be co-managed by the government and local communities ensuring environmental sustainability in Sri Lanka and beyond.		
Peña-Puch et al.	Exploring governance challenges in coastal communities through key informant perceptions in Campeche, Mexico	Campeche's coastal environment and its natural protected areas (NPA) possess great biodiversity. However, anthropogenic activities, excessive resource use and coastal ecosystems' depletion have taken their toll on ecological settings and altered communities' livelihoods. This paper describes how key informants perceive local governance's role in coastal ecosystem health. We applied semi-structured interviews (n = 17) to key informants from artisanal fisheries, community tourism projects, and natural resources conservation programs in three socio-ecological systems (SES). Most stakeholders belong cooperative societies (76.5%). While family members comprise both Isla Arena-RCBR SES 1 and Isla Aguada-LTFFPA SES 3, Campeche PBR SES 2 does not follow this pattern. Cooperatives generally pursue two economic activities (52.9%): conservation and tourism, or fisheries and tourism. In SES 1 and SES 3 most cooperatives grant information to members for decision-making and apply norms in a transparent way. Those key users have skillful knowledge of natural resources conservation, and can identify or participate in ecosystem conservation projects, such as mangrove restoration. The most consistent attributes between them are: accountability and transparency in decision making, and knowing how to perceive, locate, and sustainably use high environmental value areas. However, long-term conservation goals for many SES actors remain key objectives to achieve. While all three SES acknowledge environmental change through time, such as decreased fishery capture indices and coastal erosion, plastic and glass containers pollution levels pose the most severe risk in ecosystems health. All SES key users worry about environmental changes due to excessive fishing, and this factor remains a priority in order to balance local demand. Because this is an extremely complex, multi-causal problem, coastal communities, local government, and the academic sector must work together to achieve sustainable goals.	2023	peer-reviewed
Rifai et al.	Understanding community awareness of seagrass ecosystem services for their blue carbon conservation in marine protected areas: A case study of Karimunjawa National Park	Seagrasses provide diverse ecosystem services that support communities, yet, they are among the most threatened marine habitats. Efforts to conserve and sustainably manage seagrasses are increasing, particularly amidst the era of climate change, in which they play a critical role. However, these activities often received less attention or were slow in gaining momentum partly due to a lack of societal recognition of seagrasses and their importance. Thus, in this study, we collected community perceptions of seagrass ecosystem services and threats from 391 respondents in Karimunjawa National Park (KNP), a nationally declared protected area in Indonesia. We aim to provide insights on people's recognition of seagrasses from a local scale with strict protection measures. Overall, respondents showed varying perceptions of seagrass ecosystem services, with	2023	peer-reviewed

		provisioning services more highly perceived than regulating services. The perceived level of threats was inconclusive, though, reclamation along shorelines received higher rates (leaning toward most damaging) than other threats such as natural disasters and illegal fishing activities. Sociodemographic attributes of respondents were found to influence their perceptions, with occupation as the most pronounced driving factor. The variations observed in seagrass perceptions suggest that there is a need to strengthen and/or enhance seagrass awareness and education campaigns, which is in line with the increasing demand that public perceptions matter for the conservation and sustainable management of seagrass ecosystems. We presented here the use and insights of community perceptions for seagrass blue carbon conservation in KNP, and consequently in Indonesia, where seagrass meadows are considered globally important carbon sinks.		

Supplementary Table 2. Sample data extraction and categorization employed in this study.

Authors	Type of coastal vegetation	Country of site	Stakeholder involved
Gill	saltmarsh	Australia	key respondents (e.g., staff from WCC, Sydney Water, the Department of Land and Water Conservation, and employees of BHP Steel, Port Kembla Copper, and the Port Kembla Port Corporation)
Iftekhar and Takama	mangrove	Bangladesh	110 households
Curado et al.	saltmarsh	Spain	394 respondents - population comprised those living in the city of Huelva that were 20 years of age
Switzer	mangrove	Philippines	35 non SNPS members and 15 SNPS members.
Latif	mangrove	Malaysia	137 visitors
Queiroz et al.	mangrove	Brazil	35 shellfish collectors and 45 crab collectors who were between 25 and 50 years old
Marican et al.	mangrove	Malaysia	public who live near or close to mangrove areas and non-mangrove areas
Elggren	seagrass	Sweden	170 participants from various associations
Vegh	seagrass	Spain, Sierra Leone, Mauritania, Senegal, Guinea, Cape Verde, Gambia, Guinea-Bissau	18 experts on biology, ecology, seagrass management, and fisheries, working at local to national scales
Nessa et al.	seagrass	Indonesia	19 female gleaners were identified who had more than 10 years of experience
Quevedo et al.	mangrove	Philippines	372 household heads
Sadono et al.	mangrove	Indonesia	32 people consisting of indigenous leaders, village officials, farmers, fishermen, and teenagers
Firdaus et al.	mangrove	Indonesia	respondents were 193 people, and four experts were involved in the policy scenario analysis (four experts, each representing different groups, such asfishermen, shrimp and fish farmers, NGOs, and local government)
Gayo	mangrove	Tanzania	306 respondents from Kaole, Mlingotin, Pwani-Kunduchi, and Mtongani villages, key informants (who had specific and knowledge to the study themes), social groups of 5–8 people, such as youth, women, and men
Nyangoko et al.	mangrove	Tanzania	management institutions in the villages, mangrove resource users (fishers, mangrove cutters, food vendors, and farmers), and 60 household heads
Amone-Mabuto et al.	seagrass	Mozambique	beach front residents, users, fishers, elderly people (over 60), village leader, elder, district services officer, beach management unit leader, investor, tour guide, experienced fishers
Nijamdeen et al.	mangrove	Sri Lanka	mangrove experts (government, non-government, private sector, researchers, and mangrove-fringing community members)
Peña-Puch et al.	mangrove	Mexico	17 key informants from artisanal fisheries, community tourism projects, and natural resources conservation programs
Rifai et al.	seagrass	Indonesia	391 respondents (residents)

Supplementary Table 3. Sectoral categorization of stakeholders extracted in selected publications.

**Type of sector**	**Example of data extracted from reviewed publications**
Public sector	
• Government agency representatives	"municipal officials" (LeBlanc 1997); "government agencies" (Uychiaoco et al. 2000); "government officials" (Farley et al. 2010); "government departments" (Baker et al. 2015); "town officials" (Castagno 2018); "local government officials" (Barrett et al. 2019)
• Village heads/leaders/captains	"village elders" (Othman 2014); "village heads" (Sarmin et al. 2018); "village senior" (Duangjai et al. 2013); "local leaders" (Hugé et al., 2016)
Private sector	
• Tourism-related groups	"tourists" (Medina-Pons et al. 2004); "tourism service providers" (Syukur et al. 2021); "ecotourism" (Hugé et al., 2016); "boat operators" (Landstrom, 2006)
• Privately-owned business representatives	"fishpond owners" (Kelly 1993); "shrimp farm owners" (Peneva-Reed 2011); "business interest associations" (Baker et al. 2015); "business groups" (Sugito et al. 2019); "private landowners" (Barrett et al. 2019); "local business community" (Suharti et al. 2021)
Civil society sector	
• Local communities	"local inhabitants" (Sukardjo and Yamada 1992); "villagers" (Walters 2005); "head of households" (Shuib et al. 2012); "community respondents" (Karlina et al. 2016); "local community" (Kunasekaran et al. 2018); "community members" (Utami et al. 2018)
• Non-government organizations	"nongovernmental organizations (NGOs)" (Uychiaoco et al. 2000); "NGO representatives" (Bennett and Dearden 2014); "NGO groups" (Asyari et al. 2017); "mangrove conservation assoc" (Thompson and Friess 2019); "ENGOs" (Baker et al. 2020); "non-government officials" (Begum et al. 2022)
• Indigenous people's organizations	"indigenous people" (Almasi et al. 2018); "Indigenous populations" (Sangha et al. 2019); "ethnic groups" (Teka et al. 2019); "tribe" (Barrett et al. 2019); "indigenous leaders" (Sadono et al. 2020)
• Fisherfolk's associations	"fishermen" (Kovacs et al. 2004); "fisherfolks" (Dahdouh-Guebas et al. 2006); "fishers" (Pramitasari et al. 2015); "fishing communities (Nwosu et al. 2016); "local shellfishermen" (Castagno 2018); "fisherfolk organizations" (Balilo et al. 2023)
• Women's associations	

• Other community-based organizations (e.g., youth, religion-based, occupation-based)	“women” (Malleret-King 1998); “women’s group” (Fitriana 2014); “women who collected shellfish” (Tebaiy 2016); “housewives assoc” (Thompson and Friess 2019); “wives of members of…” (Purwanti et al. 2020); “female gleaners” (Nessa et al. 2020)<br>“social groups” (Medina-Pons et al. 2004); “cooperatives” (Hastuti and Yuliati 2017); “representatives of local organization” (Treephan et al. 2019); “Community Groups” (Purwanti et al. 2020); “village-level socio-political groups” (Carrie et al. 2022); “Community Based organizations” (Ahmed et al. 2023)
Not specified	“participants” (Kithiia 2015)<br>“concerned stakeholders” (Kim 2016)<br>“various stakeholders” (Lillebo et al. 2017)<br>“respondents” (Sulaiman et al. 2019)<br>“stakeholders representing diverse viewpoints” (Arumugam et al. 2020)

Supplementary Figure 1. Geographical distribution of studies involving perceptions of coastal vegetated ecosystems. Each country is represented by a pie chart displaying the presence or absence of themes. The size of the pie chart indicates the publication frequency.

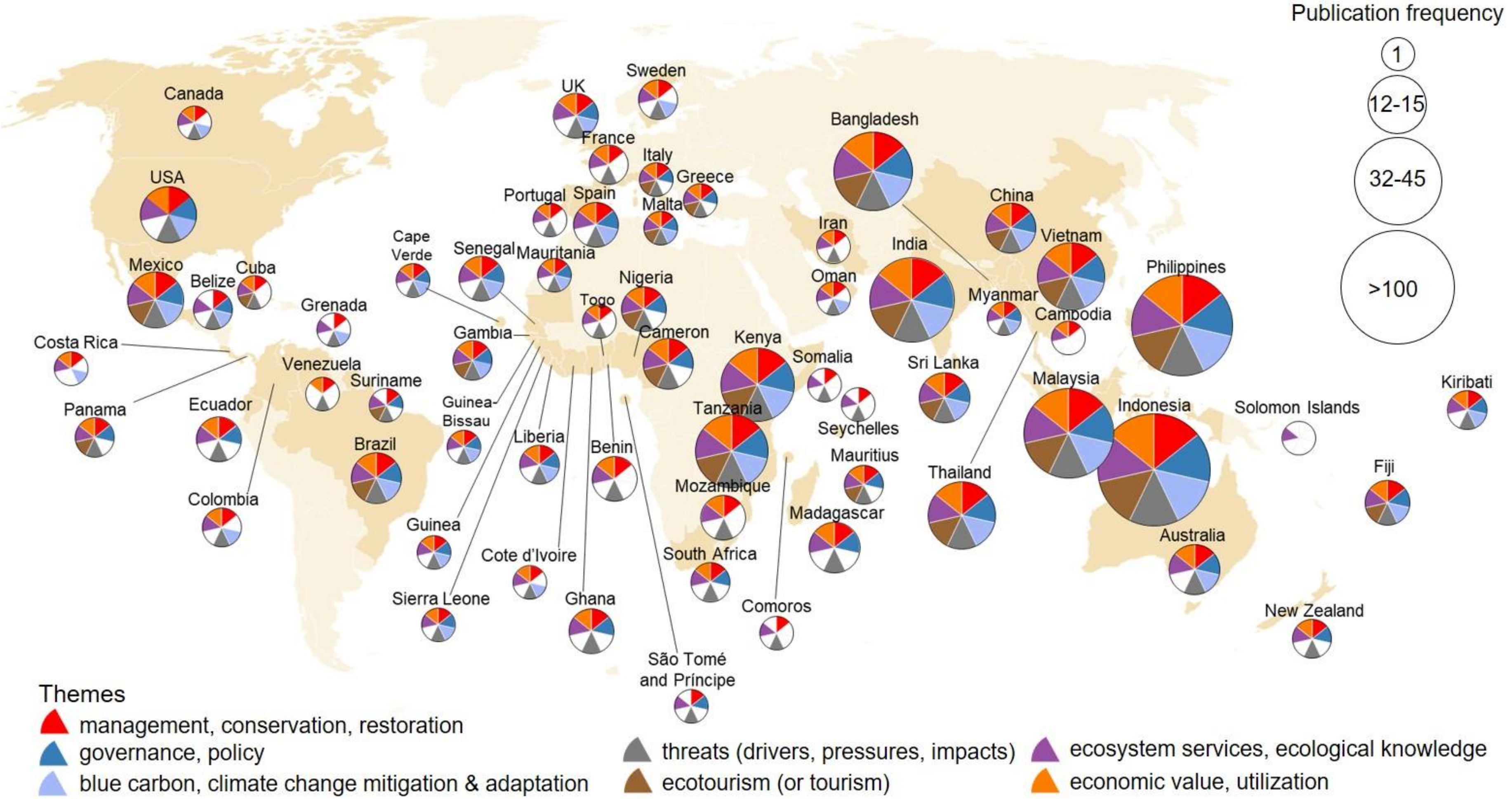

References

Ahmed, J., Kathambi, B., & Kibugi, R. (2023). Policy perspective on governance standards setting using community participation for sustainable mangrove management in Lamu Kenya. International Journal of Conservation Science, 14(1).

Almasi, M., Milow, P., & Zakaria, R. M. (2018). Participatory mangrove forest management in the Carey Island, Malaysia. Ukrainian Journal of Ecology, 8(3), 328-339.

Ambo-Rappe, R., La Nafie, Y. A., Marimba, A. A., Cullen-Unsworth, L. C., & Unsworth, R. K. (2019, November). Perspectives on seagrass ecosystem services from a coastal community. In IOP Conference Series: Earth and Environmental Science (Vol. 370, No. 1, p. 012022). IOP Publishing.

Amone-Mabuto, M., Mubai, M., Bandeira, S., Shalli, M. S., Adams, J. B., Lugendo, B. R., & Hollander, J. (2023). Coastal community's perceptions on the role of seagrass ecosystems for coastal protection and implications for management. Ocean & Coastal Management, 244, 106811.

Arumugam, M., Niyomugabo, R., Dahdouh-Guebas, F., & Hugé, J. (2021). The perceptions of stakeholders on current management of mangroves in the Sine-Saloum Delta, Senegal. Estuarine, Coastal and Shelf Science, 248, 107160.

Asante, W. A., Acheampong, E., Boateng, K., & Adda, J. (2017). The implications of land tenure and ownership regimes on sustainable mangrove management and conservation in two Ramsar sites in Ghana. Forest Policy and Economics, 85, 65-75.

Asyari, M., & RAYES, M. L. (2017). Management policy formulation of Teluk Kelumpang Natural Reserve related with mangrove forest degradation at South Borneo, Indonesia. International Journal of Conservation Science, 8(1).

Baker, S., Ayala-Orozco, B., & García-Frapolli, E. (2020). Hybrid, public and private environmental governance: the case of sustainable coastal zone management in Quintana Roo, Mexico. International Journal of Sustainable Development & World Ecology, 27(7), 625-637.

Baker, S., Paddock, J., Smith, A. M., Unsworth, R. K., Cullen-Unsworth, L. C., & Hertler, H. (2015). An ecosystems perspective for food security in the Caribbean: Seagrass meadows in the Turks and Caicos Islands. Ecosystem Services, 11, 12-21.

Balilo Jr, B. B., Candelaria, A. P., & Dioneda Sr, R. R. (2023). Assessment and Perceived Impact of Coastal Resource Management (CRM) Programs in the Southern Part of Masbate Island, Philippines. Journal of Geoscience and Environment Protection, 11(3), 87-103.

Barrett, P., Kurian, P., Simmonds, N., & Cretney, R. (2019). Community participation in the development of the Ōngātoro/Maketū Estuary project: The socio-ecological dimensions of restoring an interconnected ecosystem. Aquatic Conservation: Marine and Freshwater Ecosystems, 29(9), 1547-1560.

Basyuni, M., Bimantara, Y., Siagian, M., Wati, R., Slamet, B., Sulistiyono, N., ... & Leidonad, R. (2018, March). Developing community-based mangrove management through eco-tourism in North Sumatra, Indonesia. In IOP Conference Series: Earth and Environmental Science (Vol. 126, No. 1, p. 012109). IOP Publishing.

Begum, F., de Bruyn, L. L., Kristiansen, P., & Islam, M. A. (2022). Forest co-management in the Sundarban mangrove forest: Impacts of women's participation on their livelihoods and sustainable forest resource conservation. Environmental Development, 43, 100731.

Bennett, N. J., & Dearden, P. (2014). Why local people do not support conservation: Community perceptions of marine protected area livelihood impacts, governance and management in Thailand. Marine policy, 44, 107-116.

Cann, K. D. (2018). Untangling the Roots of the Mangrove Tree: Seeking Successful Co-Management of a Coastal/Marine Protected Area in Panama (Doctoral dissertation, The George Washington University).

Carrie, R. H., Stringer, L. C., Van Hue, L. T., Quang, N. H., Van Tan, D., Hackney, C. R., ... & Quinn, C. H. (2022). Social differences in spatial perspectives about local benefits from rehabilitated mangroves: insights from Vietnam. Ecosystems and People, 18(1), 378-396.

Castagno, K. A. (2018). Salt marsh restoration and the shellfishing industry: Co-evaluation of success components. Coastal management, 46(4), 297-315.

Curado, G., Manzano-Arrondo, V., Figueroa, E., & Castillo, J. M. (2014). Public perceptions and uses of natural and restored salt marshes. Landscape Research, 39(6), 668-679.

Dahdouh-Guebas, F., Collin, S., Lo Seen, D., Rönnbäck, P., Depommier, D., Ravishankar, T., & Koedam, N. (2006). Analysing ethnobotanical and fishery-related importance of mangroves of the East-Godavari Delta (Andhra Pradesh, India) for conservation and management purposes. Journal of Ethnobiology and Ethnomedicine, 2, 1-22.

Damastuti, E., van Wesenbeeck, B. K., Leemans, R., de Groot, R. S., & Silvius, M. J. (2023). Effectiveness of community-based mangrove management for coastal protection: A case study from Central Java, Indonesia. Ocean & Coastal Management, 238, 106498.

del Carmen Peña-Puch, A., Rivera-Arriaga, E., & Williams-Beck, L. (2023). Exploring governance challenges in coastal communities through key informant perceptions in Campeche, Mexico. Ocean & Coastal Management, 242, 106722.

de Souza Queiroz, L., Rossi, S., Calvet-Mir, L., Ruiz-Mallén, I., García-Betorz, S., Salvà-Prat, J., & de Andrade Meireles, A. J. (2017). Neglected ecosystem services: Highlighting the socio-cultural perception of mangroves in decision-making processes. Ecosystem Services, 26, 137-145.

Duangjai, W., Ngamniyom, A., Silprasit, K., & Kroeksakul, P. (2013). The guideline development for sustainable livelihood indicators of village marginal mangrove forest in the Satun Province, Thailand. Asian Social Science, 9(9), 123.

Elggren, S. (2019). People's perception of seagrass ecosystems-a Step Zero analysis in establishing marine protection of seagrass around Gotland, Sweden.

Farley, J., Batker, D., De la Torre, I., & Hudspeth, T. (2010). Conserving mangrove ecosystems in the Philippines: transcending disciplinary and institutional borders. Environmental management, 45, 39-51.

Firdaus, M., Hatanaka, K., & Saville, R. (2021). Mangrove Forest Restoration by Fisheries Communities in Lampung Bay: A study based on perceptions, willingness to pay, and management strategy. Forest and Society, 5(2), 224-244.

Fitriana, R. (2014). Assessing the impact of a marine protected area on coastal livelihoods: A case study from Pantar Island, Indonesia. Charles Darwin University (Australia).

Floor, J. R., van Koppen, C. K., & van Tatenhove, J. P. (2018). Science, uncertainty and changing storylines in nature restoration: The case of seagrass restoration in the Dutch Wadden Sea. Ocean & coastal management, 157, 227-236.

Gayo, L. (2022). Local community perception on the State Governance of mangroves in Western Indian coast of Kinondoni and Bagamoyo, Tanzania. Global Ecology and Conservation, 39, e02287.

Getzner, M., & Islam, M. S. (2013). Natural resources, livelihoods, and reserve management: a case study from Sundarbans mangrove forests, Bangladesh. International Journal of Sustainable Development and Planning, 8(1), 75-87.

Gill, N. (2005). Slag, steel and swamp: Perceptions of restoration of an urban coastal saltmarsh. Ecological Management & Restoration, 6(2), 85-93.

Harzing 2007. Publish or perish. Available from: https://harzing.com/resources/publish-or-perish .

Hastuti, T. K., & Yuliati, U. (2018). A model for mangrove forest management based on community empowerment in Bantul Regency. Journal of Environmental Management and Tourism, 8(6), 1232-1237.

Hugé, J., Velde, K. V., Benitez-Capistros, F., Japay, J. H., Satyanarayana, B., Ishak, M. N., ... & Dahdouh-Guebas, F. (2016). Mapping discourses using Q methodology in Matang mangrove forest, Malaysia. Journal of Environmental Management, 183, 988-997.

Iftekhar, M. S., & Takama, T. (2008). Perceptions of biodiversity, environmental services, and conservation of planted mangroves: a case study on Nijhum Dwip Island, Bangladesh. Wetlands Ecology and Management, 16, 119-137.

Karlina, E., Kusmana, C., Marimin, & Bismark, M. (2016). Analysis of Sustainability of Mangrove Protection Forest Management in Batu Ampar, Kubu Raya Regency, West Kalimantan Province. **Jurnal Analisis Kebijakan Kehutanan**, 13, 201–219

Kelly, P. F. (1993). Development as degradation: aquaculture, mangrove deforestation and entitlements in Batan, Philippines.

Kithiia, J. (2015). Resourceless victims or resourceful collectives: Addressing the impacts of climate change through social capital in fringing coastal communities. Ocean & Coastal Management, 106, 110-117.

Kim, J. (2016). Cangio Mangrove Forest Management: From a Perspective of Sustainable Development.

Kovacs, J. M., Malczewski, J., & Flores-Verdugo, F. (2004). Examining local ecological knowledge of hurricane impacts in a mangrove forest using an analytical hierarchy process (AHP) approach. Journal of coastal research, 20(3), 792-800.

Kunasekaran, P., Rozak, N. I. N., Adam, S. M., & Shuib, A. (2018). Perception of local communities on the indicators of governance in Tanjung Piai National Park. International Journal of Business & Society, 19.

Landstrom, I. (2006). Towards Collaborative Coastal Management in Sri Lanka?: A study of Special Area Management planning in Sri Lanka's coastal region (Doctoral dissertation, Kulturgeografiska institutionen).

Latif, F. L. B. A. (2016). Visitor perception on the potential of Matang Mangrove Forest Reserve for ecotourism.

LeBlanc, S. (1997). Gleaning in Bais Bay: a case study on an informal sector coastal activity in the Philippines.

Lillebø, A. I., Stålnacke, P., Gooch, G. D., Krysanova, V., & Bielecka, M. (2017). Pan-European management of coastal lagoons: A science-policy-stakeholder interface perspective. Estuarine, Coastal and Shelf Science, 198, 648-656.

Lukman, K. M., Uchiyama, Y., Quevedo, J. M. D., Harding, D., & Kohsaka, R. (2021). Land use changes assessment using a triangulated framework: Perception interviews, land-use/land cover observation, and spatial planning analysis in Tanjung Batu and Derawan Island, Indonesia. Human Ecology, 49(5), 551-564.

Malleret-King, D. (1998). Benefits of the Kisite marine national park as perceived by stakeholders. In Partnership for Conservation: Report of the Regional Workshop on Marine Protected Areas, Tourism, and Communities, Diani Beach, Kenya, 11-13 May, 1998 (p. 52). IUCN Eastern Africa Regional Office.

Marican, N. W., Nawi, N. M., Kamarulzaman, N. H., & Samdin, Z. (2018). Public perception towards sustainable mangrove forest programs in Malaysia. Journal of Sustainability Science and Management, 13(1), 189-199.

Medina-Pons, F. J., Terrados, J., & Tábara Villalba, D. (2004). Assessment of the perception of Majorcan society of the ecological and economical values of Posidonia oceanica (L. Delile) seagrass beds.

Mondal, B., Sarkar, N. C., Mondal, C. K., Maiti, R. K., & Rodriguez, H. G. (2012). Mangrove plants and traditional Ayurvedic practitioners in Sundarbans region of West Bengal, India. Research on Crops, 13(2), 669-674.

Mustain, M., & Leonard, R. (2019). Coastal zone management: the field approach to Wonorejo-East Surabaya. International Journal on Engineering Applications (IREA), 7(4), 145-151.

Nessa, N., Gatta, R., Ambo-Rappe, R., Jompa, J., & Yahya, A. F. (2020, September). The role of women in the utilization of Enhalus acoroides: livelihoods, food security, impacts and implications for coastal area management. In IOP Conference Series: Earth and Environmental Science (Vol. 564, No. 1, p. 012073). IOP Publishing.

Nijamdeen, T. W. G. F. M., Ratsimbazafy, H. A., Kodikara, K. A. S., Nijamdeen, T. A., Thajudeen, T., Peruzzo, S., ... & Hugé, J. (2024). Delineating expert mangrove stakeholder perceptions and attitudes towards mangrove management in Sri Lanka using Q methodology. Environmental Science & Policy, 151, 103632.

Nwosu, F. M., Otogo, G. A., Anwana, E. D., & Akpan, E. (2016). Climate Change Adaptation By Mangrove-Dependent Fishing Communities in Southern Cross River State, Nigeria. International Journal of Ecosystems and Ecology Science, 6, 139-142.

Nyangoko, B. P., Berg, H., Mangora, M. M., Shalli, M. S., & Gullström, M. (2022). Local perceptions of changes in mangrove ecosystem services and their implications for livelihoods and management in the Rufiji Delta, Tanzania. Ocean & Coastal Management, 219, 106065.

Othman, W. J. (2014). Understanding the complexity and dynamics of mangrove social-ecological systems through the use of a resilience approach in Unguja, Zanzibar (Doctoral dissertation, University of Greenwich).

Peneva-Reed, E. I. (2011). Linking local knowledge and satellite-derived land-use/land-cover change information in Krabi province, Thailand. University of South Carolina.

Pramitasari, S. D., Gallardo, W. G., & Ebbers, T. (2015). Fishers perception and attitude toward local knowledge and local practices and its role in the fisheries management: a case study in mae klong river, samut songkhram, Thailand. Turkish Journal of Fisheries and Aquatic Sciences, 15(4), 795-804.

Purwanti, P., Susilo, E., & Indrayani, E. (2020). Sustainable Management Models for Mangrove Forests Through Institutional Strengthening and the Development of Productive Business. International Journal of Social Ecology and Sustainable Development (IJSESD), 11(4), 70-81.

Quevedo, J. M. D., Uchiyama, Y., & Kohsaka, R. (2020). Perceptions of local communities on mangrove forests, their services and management: Implications for Eco-DRR and blue carbon management for Eastern Samar, Philippines. Journal of Forest Research, 25(1), 1-11.

Richerzhagen, C., Rodríguez de Francisco, J. C., Weinsheimer, F., Döhnert, A., Kleiner, L., Mayer, M., ... & Philipp, E. (2019). Ecosystem-based adaptation projects, more than just adaptation: analysis of social benefits and costs in Colombia. International journal of environmental research and public health, 16(21), 4248.

Rifai, H., Quevedo, J. M. D., Lukman, K. M., Hernawan, U. E., Alifatri, L. O., Risandi, J., ... & Kohsaka, R. (2023). Understanding community awareness of seagrass ecosystem services for their blue carbon conservation in marine protected areas: A case study of Karimunjawa National Park. Ecological Research, 38(4), 541-556.

Rivera, R., & Newkirk, G. F. (1997). Power from the people: a documentation of non-governmental organizations' experience in community-based coastal resource management in the Philippines. Ocean & Coastal Management, 36(1-3), 73-95.

Sadono, R., Soeprijadi, D., Susanti, A., Matatula, J., Pujiono, E., Idris, F., & Wirabuana, P. Y. A. P. (2020). Local indigenous strategy to rehabilitate and conserve mangrove ecosystem in the southeastern Gulf of Kupang, East Nusa Tenggara, Indonesia. Biodiversitas Journal of Biological Diversity, 21(3).

Sangha, K. K., Maynard, S., Pearson, J., Dobriyal, P., Badola, R., & Hussain, S. A. (2019). Recognising the role of local and Indigenous communities in managing natural resources for the greater public benefit: Case studies from Asia and Oceania region. Ecosystem Services, 39, 100991.

Sarmin, N. S., Ismail, M. H., Zaki, P. H., & Awang, K. W. (2018, November). Local community’s perception of mangrove change impact on their socioeconomic condition in Johor, Malaysia. In IOP Conference Series: Earth and Environmental Science (Vol. 187, No. 1, p. 012080). IOP Publishing.

Shuib, A. H. M. A. D., Yee, L. S., & Edman, S. A. L. B. I. A. H. (2012). Attitudes of local communities towards conservation of the mangrove ecosystem in Kuching, Sarawak. Malaysian Forester, 75(2), 135-146.

Sondita, M. F. A., Sunuddin, A., Hestirianoto, T., Darmansyah, S., Kamal, M. M., Munib, A. A., ... & Arkham, M. N. (2020). Community leader perspectives on the utilization of seagrass ecosystem for marine tourism in Toli-toli, Central Sulawesi, Indonesia. In IOP Conference Series: Earth and Environmental Science (Vol. 420, No. 1, p. 012029). IOP Publishing.

Sugito, T., Sulaiman, A. I., Sabiq, A., Faozanudin, M., & Kuncoro, B. Community empowerment model of coastal border based on ecotourism Model pemberdayaan masyarakat perbatasan pantai berbasis ekowisata.

Suharti, S., Andadari, L., Yeny, I., Yuniati, D., & Agustarini, R. (2021, February). Vague property status and future risk of mangroves: lesson learned from South Sulawesi, Indonesia. In IOP Conference Series: Earth and Environmental Science (Vol. 648, No. 1, p. 012047). IOP Publishing.

Sukardjo, S., & Yamada, I. (1992). The management problems and research needs of the mangrove forest in the Cimanuk delta complex, Ujung Indramayu, West Java. Japanese Journal of Southeast Asian Studies, 29(4), 468-485.

Sulaiman, B., Bambang, A. N., Purnaweni, H., Lutfi, M., & Mohammed, E. M. A. (2019). Coastal community perception of mangroves in Suli subdistrict, Luwu. Jurnal Pendidikan IPA Indonesia, 8(4), 561-569.

Switzer, S. (2015). TAKING STOCK: Community perception of a mangrove restoration and alternative livelihood program in the Verde Island Passage, Philippines.

Syukur, A., Idrus, A. A., Dewi, K. R., & Juniati, N. (2021, November). The Evidence of Seagrass Environmental Support for Local People's Economic on the South Coast of Lombok Island. In IOP Conference Series: Earth and Environmental Science (Vol. 913, No. 1, p. 012051). IOP Publishing.

Tebaiy, S. (2016). Connectivity Pattern of Socio-Ecology System of Youtefa Bay Community in Utilizing Seagrass Ecosystem. KnE Social Sciences, 44-54.

Teka, O., Houessou, L. G., Djossa, B. A., Bachmann, Y., Oumorou, M., & Sinsin, B. (2019). Mangroves in Benin, West Africa: threats, uses and conservation opportunities. Environment, development and sustainability, 21, 1153-1169.

Thompson, B. S., & Friess, D. A. (2019). Stakeholder preferences for payments for ecosystem services (PES) versus other environmental management approaches for mangrove forests. Journal of environmental management, 233, 636-648.

Treephan, P., Visuthismajarn, P., & Isaramalai, S. A. (2019). A model of participatory community-based ecotourism and mangrove forest conservation in Ban Hua Thang, Thailand. African Journal of Hospitality, Tourism and Leisure, 8(5), 1-8.

Tu, C., Ma, H., Li, Y., Fu, C., You, Z. J., Newton, A., & Luo, Y. (2022). Transdisciplinary, Co-designed and adaptive management for the sustainable development of rongcheng, a coastal city in China in the context of human activities and climate change. Frontiers in Environmental Science, 10.

Utami, N. D. N., Susiloningtyas, D., & Handayani, T. (2018, April). Community perception and participation of mangrove ecosystem in Ngurah Rai Forest Park Bali, Indonesia. In IOP Conference Series: Earth and Environmental Science (Vol. 145, No. 1, p. 012147). IOP Publishing.

Uychiaoco, A. J., Alino, P. M., & Dantis, A. L. (2000). Initiatives in Philippine coastal management: an overview. Coastal Management, 28(1), 55-63.

Vegh, T., & Potouroglou, M. (2019). High Level Assessment of Seagrass Ecosystem Services in West Africa-Perception of Stakeholders.

Vlachopoulou, E. I., Wilson, A. M., & Miliou, A. (2013). Disconnects in EU and Greek fishery policies and practices in the eastern Aegean Sea and impacts on Posidonia oceanica meadows. Ocean & coastal management, 76, 105-113.

Walters, B. B. (2005). Patterns of local wood use and cutting of Philippine mangrove forests. Economic Botany, 59(1), 66-76.

Wood, N., & Lavery, P. (2000). Monitoring seagrass ecosystem health—the role of perception in defining health and indicators. Ecosystem Health, 6(2), 134-148.

York, P. H., Smith, T. M., Coles, R. G., McKenna, S. A., Connolly, R. M., Irving, A. D., ... & Whitehead, S. (2017). Identifying knowledge gaps in seagrass research and management: an Australian perspective. Marine environmental research, 127, 163-172.
